# Therapeutic benefits of maintaining CDK4/6 inhibitors and incorporating CDK2 inhibitors beyond progression in breast cancer

**DOI:** 10.1101/2024.11.11.623139

**Published:** 2024-11-15

**Authors:** Jessica Armand, Sungsoo Kim, Kibum Kim, Eugene Son, Minah Kim, Hee Won Yang

**Affiliations:** 1 Department of Pathology and Cell Biology, Columbia University Irving Medical Center, New York, NY, 10032, USA; 2 Herbert Irving Comprehensive Cancer Center, Columbia University Irving Medical Center, New York, NY, 10032, USA

## Abstract

The combination of CDK4/6 inhibitors (CDK4/6i) and endocrine therapy has revolutionized treatment for hormone receptor-positive (HR+) metastatic breast cancer. However, the emergence of resistance in most patients often leads to treatment discontinuation with no consensus on effective second-line therapies. The therapeutic benefits of maintaining CDK4/6i or incorporating CDK2 inhibitors (CDK2i) after disease progression remain unclear. Here, we demonstrate that sustained CDK4/6i therapy, either alone or combined with CDK2i, significantly suppresses the growth of drug-resistant HR^+^ breast cancer. Continued CDK4/6i treatment induces a non-canonical pathway for retinoblastoma protein (Rb) inactivation via post-translational degradation, resulting in diminished E2F activity and delayed G1 progression. Importantly, our data highlight that CDK2i should be combined with CDK4/6i to effectively suppress CDK2 activity and overcome resistance. We also identify cyclin E overexpression as a key driver of resistance to CDK4/6 and CDK2 inhibition. These findings provide crucial insights into overcoming resistance in HR^+^ breast cancer, supporting the continued use of CDK4/6i and the strategic incorporation of CDK2i to improve therapeutic outcomes.

## Introduction

Metastatic breast cancer remains a leading cause of cancer-related mortality in women globally (1,2). A key dysregulation in breast cancer involves the overactivation of cyclin-dependent kinases 4 and 6 (CDK4/6) (3–5). Active CDK4/6 phosphorylates the retinoblastoma protein (Rb), a crucial regulator that prevents cell-cycle initiation by sequestering E2F transcription factors (6). Rb phosphorylation results in the release of E2F, thereby promoting CDK2 activation and cell proliferation (7,8). Understanding this mechanism has driven significant advancements in therapeutic strategies, particularly for hormone receptor-positive (HR^+^)/human epidermal growth factor receptor 2-negative (HER2^-^) breast cancer, which constitutes approximately 70% of breast cancer cases (9). The current standard first-line treatment for HR^+^/HER2^−^ metastatic breast cancer is a combination of CDK4/6 inhibitors (CDK4/6i) and endocrine therapy (ET) (4,5,10). Although this approach has markedly improved patient outcomes, resistance remains a significant challenge, with about 30% of patients developing resistance within two years and most eventually facing resistance (11,12). Upon disease progression, CDK4/6i therapy is often discontinued, leading to aggressive tumor growth with effective second-line therapies lacking.

While continuing ET after disease progression has demonstrated clinical benefits (13–17), the efficacy of maintaining CDK4/6i treatment post-progression is still elusive. Ongoing clinical trials investigate the potential advantages of continuing CDK4/6i therapy using FDA-approved inhibitors such as palbociclib, abemaciclib, and ribociclib in patients experiencing disease progression (18–22). The MAINTAIN phase 2 trial suggests that continuing ribociclib with alternative ET post-progression on a palbociclib and ET regimen can significantly extend progression-free survival (PFS) compared to switching to ET alone (22). Similarly, interim results from the postMONARCH phase 3 study indicate that adding abemaciclib to ET improved PFS compared to ET alone in patients who have progressed on ET and a prior CDK4/6i (23). Conversely, the PACE and PALMIRA clinical trials did not observe a significant PFS benefit when maintaining palbociclib combined with new ET after disease progression (19,24).

Like CDK4/6, CDK2 also phosphorylates Rb before the G1/S transition (7,25). The relevance of CDK2 activation in CDK4/6i-resistant tumors highlights the potential of targeting CDK2 to overcome CDK4/6i resistance (26–30). Several clinical trials are exploring CDK2 inhibitors (CDK2i) in patients who have progressed on CDK4/6i-based treatments as monotherapies or in combination with CDK4/6i. Determining the optimal strategy for incorporating CDK2i in treating HR^+^/HER2^−^ breast cancer that has developed drug resistance remains a critical question.

This study emphasizes that continued CDK4/6i treatment in drug-resistant cells leads to incomplete and ineffective retinoblastoma (Rb) inactivation, significantly slowing G1-phase progression. Additionally, it highlights the therapeutic potential of combining CDK2i with CDK4/6i as an effective second-line treatment strategy. Notably, the research underscores the critical role of cyclin E overexpression in driving resistance to the CDK2i and CDK4/6i combination, providing insights into overcoming such resistance in future cancer treatments.

## Results

### Maintaining CDK4/6i treatment attenuates the growth of drug-resistant cells by extending G1-phase progression.

**Figure 1.**
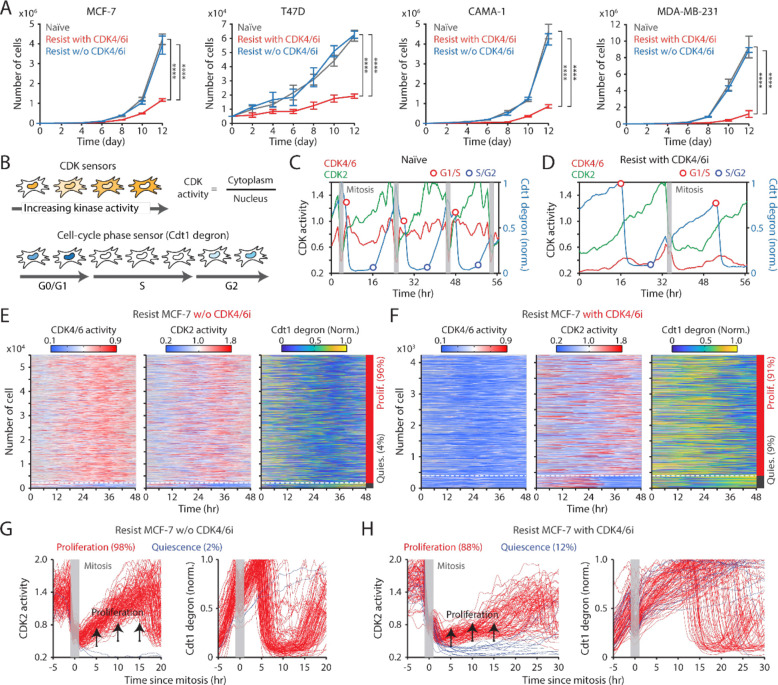
Continuous CDK4/6i treatment attenuates drug-resistant cell growth. (A) Growth curves of drug-naïve and drug-resistant cells. Palbociclib (1 μM) was either withdrawn or maintained in drug-resistant cells. Data are shown as mean ± SD (*n* = 3 biological replicates). Statistical significance was determined using one-way ANOVA with Tukey's post-hoc analysis (**** *P* < 0.0001). (B) Schematic illustration of live-cell sensors for CDK4/6 and CDK2 activities (top) and cell-cycle phase (bottom). (C-D) Representative single-cell traces showing CDK4/6 and CDK2 activities and Cdt1-degron intensity in drug-naïve (C) and drug-resistant (D) MCF-7 cells. Palbociclib (1 μM) was maintained in drug-resistant cells. Vertical gray bars indicate mitotic events, while circles mark G1/S (red) and S/G2 (blue) transitions. (E-F) Heatmaps showing single-cell traces for CDK4/6 (left) and CDK2 (middle) activities, and Cdt1-degron intensity (right) in drug-resistant cells without (E) or with (F) continuous palbociclib (1 μM) treatment. Proliferating cells were identified based on CDK2 activity (>1 for more than 2 hr between 30 and 48 hr). (G-H) Single-cell traces of CDK2 activity (left) and Cdt1-degron intensity (right) aligned by the end of mitosis (anaphase) in drug-resistant cells without (G) or with (H) continuous palbociclib (1 μM) treatment. Based on CDK2 activity (black line), cells were classified as either proliferation (red) or quiescent (blue). Mitosis timing is marked in gray.

To investigate the effect of maintaining CDK4/6i treatment on drug-resistant cells, we used three HR^+^/HER2^−^ breast cancer cell lines (MCF-7, T47D, and CAMA-1) and a triple-negative breast cancer cell line (MDA-MB-231). These cells were chronically exposed to palbociclib for over a month to induce drug resistance. We confirmed increased half-maximal inhibitory concentrations (IC50) for palbociclib in drug-resistant cells compared to drug-naïve cells (Figure S1A). We either maintained or discontinued CDK4/6i treatment in these drug-resistant cells and evaluated the cumulative proliferation rate over 12 days, along with drug-naïve cells. We found a significantly slower growth rate in drug-resistant cells maintained on CDK4/6i treatment compared to other conditions (Figure 1A). Conversely, drug-resistant cells with discontinued CDK4/6i treatment exhibited similar growth rates to drug-naïve cells. These results suggest that despite the development of drug resistance, maintaining CDK4/6i treatment significantly impedes the growth rate of drug-resistant cells.

We next monitored cell-cycle progression in individual cells to understand how continued CDK4/6i treatment suppresses the growth rate in drug-resistant cells. We employed MCF-7 and MDA-MB-231 cells expressing live-cell sensors for CDK4/6 (31) and CDK2 (32) activities, cell-cycle phases (Cdt1 degron) (33), and a nucleus marker (histone 2B) for individual cell tracking. The CDK sensors dynamically shuttle between the nucleus and cytoplasm based on their phosphorylation by the relevant kinase (Figure 1B top). The Cdt1 degron is degraded during the S phase, enabling visualization of the G1/S and S/G2 transitions (Figure 1B bottom). After mitosis, proliferating drug-naïve cells continuously activated CDK4/6, followed by gradual increase in CDK2 activity to enter the S phase (Figure 1C). In contrast, CDK4/6i-resistant cells activated CDK2 with low CDK4/6 activity to enter the S phase (Figure 1D). When evaluating thousands of cells, approximately 98% of drug-naïve cells continuously activated both CDK4/6 and CDK2 to enter the cell cycle (Figure S1B and S1C). While drug withdrawal led to CDK4/6 reactivation, over 90% of drug-resistant cells continued to proliferate regardless of the presence of CDK4/6i (Figure 1E, 1F, S1D, and S1E). To monitor cell-cycle dynamics, we aligned cells by the end of mitosis (cytokinesis) and classified them based on CDK2 activity into proliferating and quiescent cells. Without CDK4/6i treatment, drug-resistant cells exhibited similar kinetics of CDK4/6 and CDK2 activities, as well as S-phase entry, compared to drug-naïve cells (Figure 1G and S2A–S2D). However, with continued CDK4/6i treatment, drug-resistant cells showed low CDK4/6 activity, slower activation kinetics of CDK2, and increased heterogeneity in the timing of S-phase entry, as indicated by Cdt1 degradation, compared to other conditions (Figure 1H, S2E, and S2F). These results indicate that continuous treatment with CDK4/6i leads to marked deceleration and irregularity in cell-cycle progression of drug-resistant cells, thus suppressing their growth rates.

CDK6 overexpression has emerged as a key driver of CDK4/6i resistance (34–37). In line with these findings, we observed elevated CDK6 levels in CDK4/6i-resistant cells (Figure S3A). To investigate the role of CDK6 in modulating CDK4/6 activity under resistance, we established CDK6 knockout (KO) MCF-7 cells using CRISPR-Cas9 and induced resistance to CDK4/6i (Figure S3B). Despite CDK6 depletion, CDK4/6 activity remained comparable between wild-type and CDK6-KO cells under continuous CDK4/6i treatment (Figure S3C–S3F). These results suggest a potential non-canonical role for CDK6 in mediating resistance to CDK4/6i therapy.

**Figure 2.**
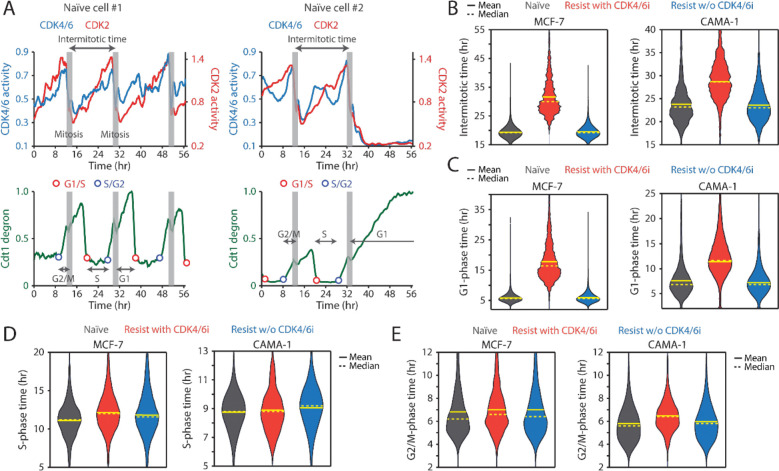
CDK4/6i maintenance extends G1-phase progression. (A) Representative single-cell traces showing CDK4/6 and CDK2 activities (top) and Cdt1-degron intensity (bottom) in drug-naïve MCF-7 cells. (B-E) Violin plots showing intermitotic time (*n* > 600 cells/condition) (B), G1-phase duration (*n* > 2,000 cells/condition) (C), S-phase duration (*n* > 400 cells/condition) (D), and G2/M-phase duration (*n* > 250 cells/condition) (E) in MCF-7 (left) and CAMA-1 (right) cells. Solid and dashed yellow lines indicate mean and median, respectively.

To assess the impact of continuous CDK4/6i treatment on cell-cycle progression, we measured the intermitotic time and the duration of each cell-cycle phase in MCF-7 and CAMA-1 cells (Figure 2A). The intermitotic time was comparable between drug-naïve and drug-resistant cells without ongoing CDK4/6i treatment (Figure 2B). However, in drug-resistant cells that were continuously treated with CDK4/6i, the intermitotic time was extended by 30–50% compared to other conditions. Moreover, continuous CDK4/6i treatment prolonged the G1 phase by approximately 200–300% without affecting the duration of the S and G2/M phases (Figure 2C–2E). Our data indicate that maintaining CDK4/6i treatment in drug-resistant cells significantly extends the G1 phase, thereby decelerating the overall growth rate.

### Maintaining CDK4/6i treatment triggers an ineffective Rb inactivation pathway.

**Figure 3.**
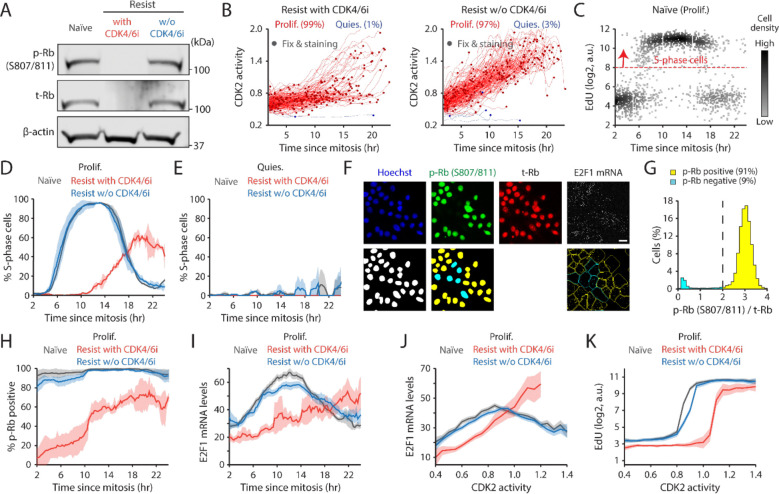
CDK4/6i maintenance triggers an ineffective Rb inactivation pathway. (A) Immunoblot showing phosphorylated Rb (S807/811) (p-Rb), total Rb (t-Rb), and β-actin protein levels in MCF-7 cells. Drug-resistant cells were harvested two weeks after drug withdrawal. (B) Singe-cell traces showing CDK2 activity aligned by mitosis in proliferating (red) and quiescent (blue) MCF-7 cells. Circles indicate the time of fixation and staining (*n* = 200 cells). (C) Scatter plot of EdU intensity classified by time since mitosis in drug-naïve cells. The red dotted line marks the threshold for S phase (*n* = 2,000 cells). (D-E) Percentage of S-phase cells as a function of time since mitosis in proliferating (D) and quiescent (E) cells. Data are shown as mean ± SD (*n* = 2 biological replicates). (F) Representative immunostaining of Hoechst, p-Rb, t-Rb, and E2F1 mRNA FISH (top). Processed images that show nucleus segmentation, p-Rb classification, and mRNA puncta detection (bottom). (G) Histogram showing the percentage of cells with p-Rb normalized by t-Rb. (H-I) Percentage of cells with p-Rb (H) and E2F1 mRNA levels (I) as a function of time since mitosis. Data are shown as mean ± SD (*n* = 3 biological replicates) (H) or mean ± 95% confidence intervals (*n* > 2,500 cells/condition) (I). (J-K) E2F1 mRNA (J) and EdU (K) levels as a function of CDK2 activity. Data are shown as mean ± 95% confidence intervals (*n* > 2,500 cells/condition).

We sought to elucidate the molecular mechanisms underlying the extended G1 phase and slow CDK2 activation kinetics in drug-resistant cells under continuous CDK4/6i treatment. Our recent studies demonstrated that CDK4/6 inhibition initially halts cell proliferation through Rb activation but ultimately reduces Rb protein due to decreased stability (38,39). We confirmed a decrease in both total and phosphorylated Rb levels in drug-resistant cells continuously treated with CDK4/6i compared to drug-naïve cells (Figure 3A). Discontinuation of CDK4/6i treatment restored both total and phosphorylated Rb levels. Importantly, the reduction of Rb protein in drug-resistant cells was incomplete, as evidenced by comparisons to Rb-KO cells (Figure S4A). Furthermore, Rb KO in drug-resistant cells reversed the extended G1 duration and the slowed activation kinetics of CDK2 without affecting CDK4/6 activity, even in the presence of continuous CDK4/6i treatment (Figure S4B and S4C). This led us to hypothesize that maintaining CDK4/6i treatment in drug-resistant cells induces a non-canonical pathway for Rb inactivation via its degradation. This passive Rb inactivation may result in ineffective E2F and CDK2 activation kinetics, delaying G1-phase progression.

To test our hypothesis, we monitored CDK2 activity to isolate proliferating cells and aligned them with corresponding fixed-cell data by the end of mitosis (Figure 3B and S5A). Using 5-ethynyl-2´-deoxyuridine (EdU) incorporation, we measured the kinetics of entry into the S phase. Almost all drug-naïve and -resistant cells without CDK4/6i treatment transitioned sharply into the S-phase about 6 hr after mitosis and completed DNA replication within 12 hr (Figure 3C, 3D, and S5B). However, drug-resistant cells under continued CDK4/6i treatment exhibited a dramatically slowed G1/S transition and increased variability in entering the S-phase compared to other conditions (Figure 3D and S5C). We also confirmed no S-phase entry in quiescent cells (Figure 3E). Using immunostaining and mRNA FISH, we evaluated the kinetics of Rb phosphorylation and E2F activity (Figure 3F). Cells were classified based on Rb phosphorylation at Serine 807/811, which is a previously characterized marker for hyperphosphorylation (40) (Figure 3G). The expression levels of E2F1 mRNA are regulated via autoregulatory control (41), thereby serving as a proxy of E2F transcriptional activity (7,40). In the absence of CDK4/6i, both drug-naïve and -resistant cells fully induced Rb phosphorylation before S-phase entry (Figure 3H). Conversely, in the presence of CDK4/6i, drug-resistant cells initiated CDK2 activation and G1 progression without Rb phosphorylation, showing low and slow E2F mRNA accumulation (Figure 3H and I). Moreover, when comparing CDK2 activity with levels of E2F1 mRNA or EdU incorporation, drug-resistant cells under continuous CDK4/6i treatment required higher CDK2 activity to achieve the same levels of E2F1 mRNA and to trigger the G1/S transition compared to other conditions (Figure 3J, 3K, S5D, and S5E). The observed reduction in E2F1 mRNA during the S phase could be attributed to the suppression of atypical E2Fs, E2F7 and E2F8 (42). These findings suggest that maintaining CDK4/6i treatment in drug-resistant cells induces inefficient Rb inactivation, characterized by reduced and delayed E2F activation kinetics. Such alterations substantially increase the duration of the G1 phase and the variability in the G1/S transition.

### Maintenance of CDK4/6i suppresses the growth of drug-resistant tumors.

**Figure 4.**
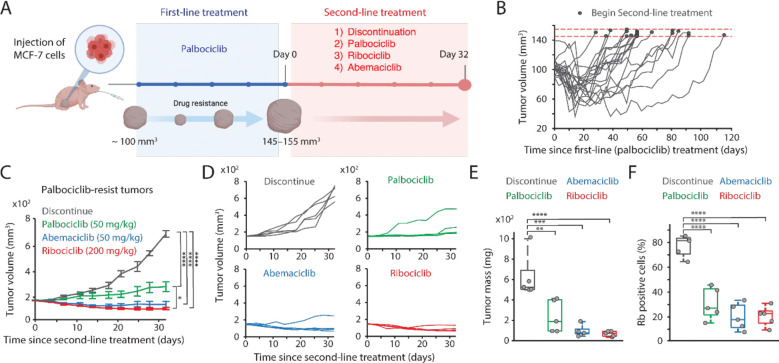
CDK4/6i maintenance suppresses the growth of drug-resistant tumors. (A) Schematic representation of experimental design. Once tumors reached a volume of 100 mm^3^, mice were treated with palbociclib. Following the development of resistance, mice were randomly assigned to one of four treatment groups: treatment discontinuation, palbociclib maintenance, switch to ribociclib, or switch to abemaciclib for 32 days. (B) Tumor growth curves showing the establishment of resistance to palbociclib. Horizontal red dotted lines (145–155 mm^3^) indicate the point at which mice were assigned to second-line treatment. (C-D) Averaged (C) and individual (D) tumor growth traces following second-line treatments. Data are shown as mean ± SEM. Statistical significance was determined using one-way ANOVA with Tukey's post-hoc analysis (* *P* < 0.05, **** *P* < 0.0001). (E-F) Box plots showing tumor mass (E) and the percentage of Rb-positive cells (F) after second-line treatment. Statistical significance was determined using one-way ANOVA with Tukey's post-hoc analysis (** *P* < 0.05, *** *P* < 0.01, **** *P* < 0.0001).

We investigated the therapeutic benefits of maintaining CDK4/6i treatment after the onset of drug resistance in vivo. To this end, we established an MCF-7 xenograft model by orthotopically inoculating drug-naïve MCF-7 cells into the mammary fat pad of immunodeficient mice. When the tumor reached a volume of 100 mm^3^, we treated mice with palbociclib and monitored tumor growth (Figure 4A). After an initial phase of tumor stasis and regression lasting for varying durations, we observed the emergence of drug resistance, evidenced by tumor regrowth (Figure 4B). Once tumors regrew to volumes between 145 and 155 mm^3^, the mice were randomly assigned to secondary treatment groups: discontinuation of drug treatment, continuation with palbociclib, or switching to ribociclib or abemaciclib. Consistent with our in vitro results, maintaining CDK4/6i significantly attenuated overall tumor growth compared to discontinuing drug treatment (Figure 4C–E). Analysis of Rb expression in tumor tissues post-secondary treatment revealed the restoration of total Rb expression exclusively in the drug-discontinued condition (Figure 4F). These data underscore the therapeutic advantages of maintaining CDK4/6i to attenuate the growth of drug-resistant tumors.

### Addition of ET augments the efficacy of CDK4/6i maintenance

Our recent studies have highlighted the pivotal role of c-Myc in amplifying E2F activity and fostering CDK4/6i resistance following alternative Rb inactivation (38,39). Using a doxycycline-inducible system, we found that induction of c-Myc significantly facilitated the growth of drug-resistant cells under ongoing CDK4/6i treatment (Figure S6A). Furthermore, despite similar low CDK4/6 activity, c-Myc induction facilitated cell-cycle progression in CDK4/6i-resistant cells (Figure S6B–D). These data demonstrated that c-Myc promotes CDK4/6i resistance by facilitating cell-cycle progression in drug-resistant cells.

**Figure 5.**
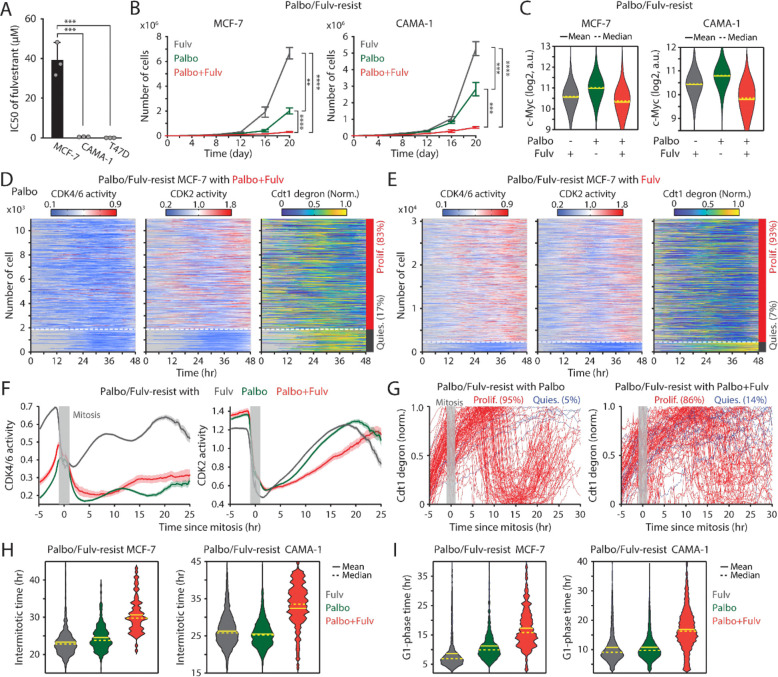
Maintaining CDK4/6i and ET synergistically suppresses the growth of drug-resistant cells. (A) IC50 values of fulvestrant. Data are shown as mean ± SD (*n* = 3 biological replicates). Statistical significance was determined using one-way ANOVA with Tukey's post-hoc analysis (*** *P* < 0.001). (B) Growth curves of MCF-7 (left) and CAMA-1 (right) cells resistant to palbociclib and fulvestrant. Cells were maintained with fulvestrant (500 nM) or palbociclib (1 μM) alone or their combination. Data are shown as mean ± SD (*n* = 3 biological replicates). Statistical significance was determined using one-way ANOVA with Tukey's post-hoc analysis (** *P* < 0.01, *** *P* < 0.001, **** *P* < 0.0001). (C) Violin plots representing c-Myc levels in cells resistant to palbociclib and fulvestrant treated with the indicated drugs for one week before fixation. Solid and dashed yellow lines represent mean and median, respectively (*n* > 2,000 cells/condition). (D-E) Heatmaps showing single-cell traces for CDK4/6 (left) and CDK2 (middle) activities and Cdt1-degron intensity (right) in drug-resistant cells maintained with palbociclib (1 μM) and fulvestrant (500 nM) (D) or fulvestrant alone (E). Proliferating cells were identified based on CDK2 activity (>1 for more than 2 hr between 30 and 48 hr). (F) Averaged traces of CDK4/6 (left) and CDK2 (right) activities aligned by mitosis in drug-resistant cells treated with the indicated drugs. Data are shown as mean ± 95% confidence intervals (*n* > 1,000 cells/condition). (G) Single-cell traces of Cdt1-degron intensity aligned by mitosis in drug-resistant cells treated with palbociclib (1 μM) (left) or palbociclib + fulvestrant (500 nM) (right). (H-I) Violin plots showing intermitotic time (*n* > 74 cells/condition) (H), and G1-phase duration (*n* > 630 cells/condition) (I) in MCF-7 (left) and CAMA-1 (right) cells. Solid and dashed yellow lines indicate mean and median, respectively.

Given the estrogen responsiveness of c-Myc (43), we hypothesized that the continued addition of ET could further suppress the growth of CDK4/6i-resistant cells by downregulating c-Myc expression. We employed the estrogen receptor antagonist fulvestrant to evaluate the therapeutic benefit of maintaining the combination of CDK4/6i and ET. While MCF-7 cells exhibited primary resistance to fulvestrant, CAMA-1 and T47D cells displayed sensitivity to this drug (Figure 5A). We used these MCF-7 and CAMA-1 cells to examine whether primary ET resistance contributes to the benefit of maintaining CDK4/6i and ET in drug-resistant cells. These cells were chronically exposed to palbociclib and fulvestrant for over two months to induce drug resistance. Subsequently, these resistant cell lines were subjected to continuous treatment with either palbociclib or fulvestrant alone or in combination, and their cumulative proliferation rate was monitored. We observed a significantly slower growth rate in drug-resistant cells continuously exposed to the combination therapy compared to other treatment conditions, not only in CAMA-1 but also in MCF-7 cells (Figure 5B, S7A, and S7B). Continued treatment with fulvestrant, either alone or combined with palbociclib, reduced c-Myc levels compared to palbociclib maintenance alone (Figure 5C). Our data indicate distinct resistance mechanisms between CDK4/6i and ET. Therefore, regardless of primary resistance to ET, the continued addition of ET to CDK4/6i treatment is beneficial in suppressing the growth of drug-resistant cells by inhibiting c-Myc.

To elucidate the mechanisms underlying the attenuation of growth resulting from continued treatment with the drug combination, we assessed CDK4/6 and CDK2 activities along with cell-cycle progression (Cdt1 degron) in drug-resistant MCF-7 cells. Cells were continuously treated with palbociclib or fulvestrant alone or in combination. By classifying cells into proliferation and quiescence based on CDK2 activation, we found over 80% of cells were proliferating in all conditions (Figure 5D, 5E, and S7C). Furthermore, we observed reactivation of CDK4/6 in drug-resistant cells continuously treated with fulvestrant alone. However, when we monitored cell-cycle dynamics by aligning cells to mitosis, the maintenance of the drug combination caused slower CDK2 activation kinetics and greater heterogeneity in S-phase entry, as indicated by Cdt1 degradation, compared to single-drug treatment conditions (Figure 5F, 5G, S7D, and S7E). By analyzing each cell-cycle duration, we observed a significant increase in intermitotic time and G1-phase duration, while S- or G2-phase durations remained relatively unchanged (Figure 5H, 5I, S7F, and S7G). This emphasizes the role of the drug combination in altering cell-cycle kinetics in the G1 phase. Our findings showed the therapeutic benefits of maintaining a combination of CDK4/6i and ET in attenuating the growth of drug-resistant cells.

### The benefit of using CDK2i in combination with CDK4/6i as a second-line therapy

**Figure 6.**
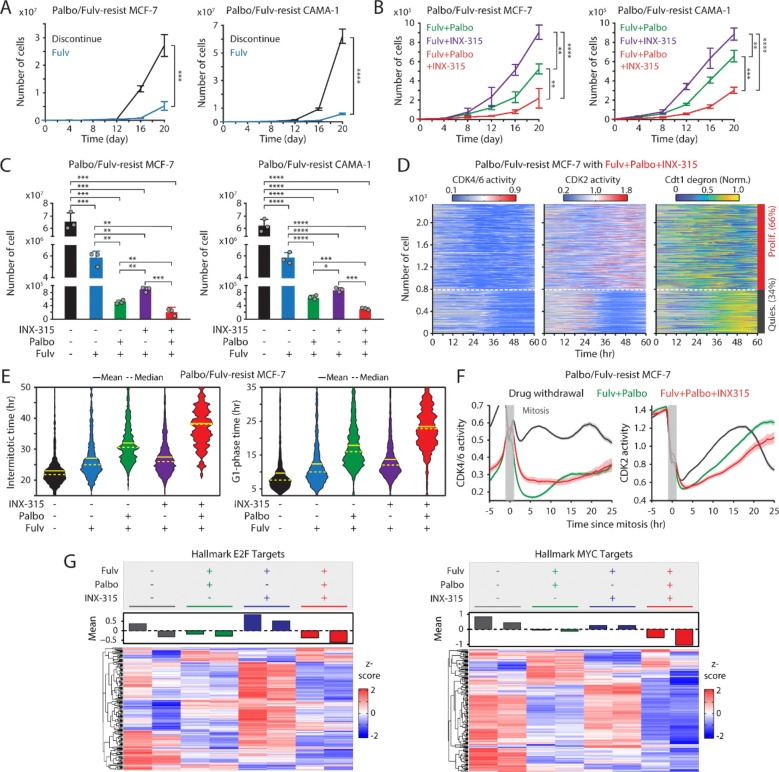
Therapeutic benefit of combining CDK2i with CDK4/6i and ET. (A-B) Growth curves of MCF-7 (left) and CAMA-1 (right) cells resistant to palbociclib and fulvestrant under various treatment conditions: drug discontinuation, fulvestrant (500 nM) alone, or in combination with palbociclib (1 μM) and/or INX-315 (100 nM). Data are shown as mean ± SD (*n* = 3 biological replicates). *P* values were calculated with an unpaired *t*-test (A) and using one-way ANOVA with Tukey's post-hoc analysis (B) (*P < 0.05, ** *P* < 0.01, *** *P* < 0.001, **** *P* < 0.0001). (C) Cell numbers 20 days after drug treatment. Data are shown as mean ± SD (*n* = 3 biological replicates). *P* values were calculated using an unpaired *t*-test (* *P* < 0.05, ** *P* < 0.01, *** *P* < 0.001, **** *P* < 0.0001). (D) Heatmaps of single-cell traces for CDK4/6 (left) and CDK2 (middle) activities, and Cdt1-degron intensity (right) in palbociclib/fulvestrant resistant MCF-7 cells treated with the triple combination of palbociclib (1 μM), fulvestrant (500 nM), and INX-315 (100 nM) for one week before imaging. Proliferating cells were identified based on CDK2 activity (>1 for more than 2 hr between 30 and 48 hr). (E) Violin plots showing intermitotic time (*n >* 200 cells/condition) (left) and G1-phase duration (*n >* 900 cells/condition) (right). Solid and dashed yellow lines indicate mean and median, respectively. (F) Averaged traces of CDK4/6 (left) and CDK2 (right) activities aligned by mitosis in drug-resistant cells treated with the indicated drugs. Data are shown as mean ± 95% confidence intervals (*n* > 1,000 cells/condition). (G) Heatmaps comparing gene expression profiles for hallmark E2F (left) and MYC (right) targets in drug-resistant MCF-7 cells treated with the indicated drugs for 20 days. Samples were collected as biological duplicates.

We next evaluated the potential of integrating CDK2i as a second-line treatment by employing the CDK2i INX-315. We examined the growth of palbociclib/fulvestrant-resistant MCF-7 and CAMA-1 cells under various treatment regimens: treatment discontinuation, fulvestrant alone, fulvestrant + palbociclib, fulvestrant + INX-315, and fulvestrant + palbociclib + INX-315. Continued treatment with the combination of palbociclib and fulvestrant significantly reduced the growth rate of drug-resistant cells compared to treatment discontinuation, fulvestrant alone, and fulvestrant + INX-315 (Figure 6A–6C). Notably, the triple combination most effectively suppressed the growth of these drug-resistant cells. We next investigated cell-cycle dynamics by assessing CDK activities and Cdt1-degron levels a week after drug treatment. Consistent with earlier results, CDK4/6 reactivation was observed following palbociclib withdrawal (Figure S8A–S8D). Furthermore, we found CDK2 reactivation even in the presence of INX-315 (Figure 6D and S8D). The triple-drug combination caused the most extended intermitotic time and G1-phase duration without affecting the S and G2 phases (Figure 6E and S8E). Analysis of cells synchronized by mitosis revealed that the triple-drug combination most prominently delayed CDK2 activation kinetics (Figure 6F). To further understand the effects of second-line therapies on the transcriptome landscape of CDK4/6i/ET-resistant cells, we performed RNA sequencing 20 days after drug treatment. In line with our previous observations, continued palbociclib and fulvestrant more effectively suppressed E2F-target gene expression compared to the INX-315 and fulvestrant combination, with the triple-drug regimen showing the most robust inhibition of both E2F and Myc target gene expression (Figure 6G and S8F). These data suggest that despite CDK2 reactivation, the addition of CDK2i to CDK4/6i and ET maintenance therapy further attenuates E2F activity and CDK2 activation kinetics, thereby inhibiting the growth rate of drug-resistant cells.

### Role of cyclin E/A in CDK2 reactivation under combined CDK4/6 and CDK2 inhibition

**Figure 7.**
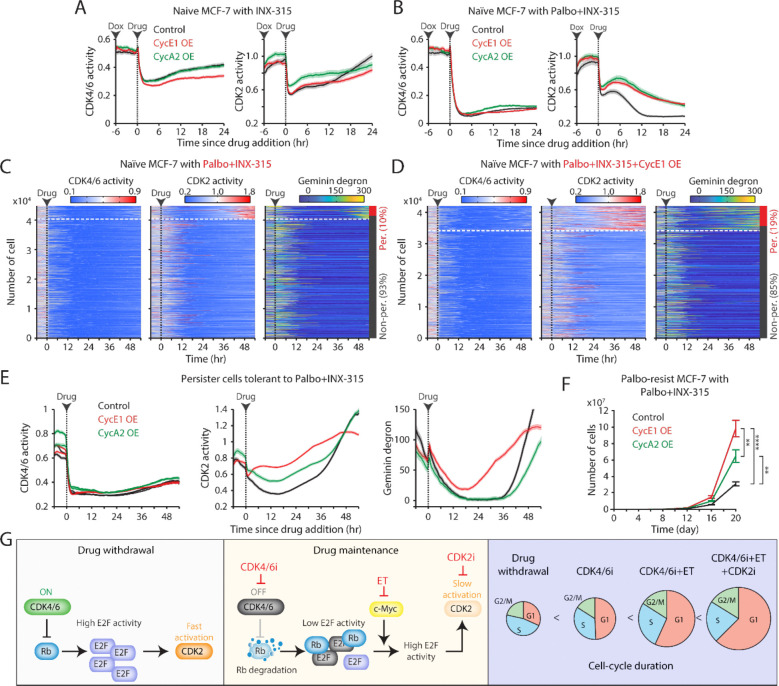
Overexpression of cyclin E and A facilitates resistance to the combination of CDK2i and CDK4/6i. (A-B) Averaged traces of CDK4/6 (left) and CDK2 (right) activities in MCF-7 cells with or without cyclin E1 or A2 overexpression. Cells were treated with doxycycline (500 nM) 6 hr before the addition of INX-315 (1 μM) alone (A) or in combination with palbociclib (1 μM) (B). Data are shown as mean ± 95% confidence intervals (*n* > 900 cells/condition). (C-D) Heatmaps of single-cell traces for CDK4/6 (left) and CDK2 (middle) activities, and Geminin-degron intensity (right) in drug-naïve MCF-7 cells without (C) or with (D) cyclin E1 overexpression. Cells were treated with palbociclib (1 μM), INX-315 (100 nM), and doxycycline (500 nM). Persister cells were identified based on CDK2 activity (>1 for more than 2 hr between 30 and 48 hr). (E) Averaged traces of CDK4/6 (left) and CDK2 (middle) activities and Geminin degron intensity (right) in persister cells tolerant to combination palbociclib (1 μM), and INX-315 (100 nM) without or with cyclin E1 or A2 overexpression. Data are shown as mean ± 95% confidence interval. *n* > 2500 cells/condition. (F) Growth curves of palbociclib-resistant MCF-7 cells without or with cyclin E1 or A2 overexpression. Cells were treated with palbociclib (1 μM), INX-315 (100 nM), and doxycycline (500 nM). Data are shown as mean ± SD (*n* = 3 biological replicates). Statistical significance was determined using one-way ANOVA with Tukey's post-hoc analysis (** *P* < 0.01, **** *P* < 0.0001). (G) Summary schematic illustrating the mechanisms underlying the benefits of continued CDK4/6i and ET therapies and the introduction of CDK2i in drug-resistant cells.

A recent study identifies the cyclin A-CDK complex as a key driver of rapid adaptation to CDK2 inhibition (44). To investigate the mechanism of CDK2 reactivation during CDK2i treatment, we explored the role of the CDK2 activators, cyclin E and A. Using a doxycycline-inducible system, we induced the overexpression of cyclin E1 or A2 in drug-naïve MCF-7 cells (Figure S9A). While treatment with INX-315 (100 nM–1 μM) initially inhibited CDK2 activity, CDK2 reactivation occurred within 1–2 hr after drug treatment, regardless of cyclin E1 or A2 overexpression (Figure 7A and S9B–S9D). The combination of CDK2i and CDK4/6i blocked this rapid CDK2 reactivation, but the overexpression of cyclin E1 or A2 hindered complete suppression (Figure 7B and S9E–S9G). These findings suggest that CDK4/6 inhibition is necessary for effectively suppressing CDK2 activity by CDK2i, and that cyclin E1 and A2 overexpression may contribute to CDK2 reactivation.

We next examined the impact of cyclin E and A overexpression in the development of persister cells, which are implicated in residual tumor growth and eventual resistance (38,45). We used drug-naïve MCF-7 cells expressing live-cell sensors for CDK2, CDK4/6, and anaphase-promoting complex/cyclosome (APC/C) (Geminin degron) activities (46). APC/C is a multi-subunit E3 ubiquitin ligase typically inactivated near the G1/S transition, leading to Geminin-degron accumulation. Treatment with CDK4/6i and CDK2i induced near-complete cell-cycle arrest within 24 hr (Figure 7C). However, approximately 10% of cells developed a persister phenotype through CDK2 reactivation. Overexpression of cyclin E1 increased the percentage of persister cells, while cyclin A2 had no obvious impact (Figure 7D and S10). Further kinetic analysis revealed that cyclin E1 overexpression accelerated CDK2 reactivation and APC/C inactivation in persister cells (Figure 7E). These findings indicate that cyclin E1, but not cyclin A2, facilitates CDK2 reactivation and persister development, promoting resistance to the CDK4/6i and CDK2i combination.

Since cyclin A is a substrate of APC/C (47), the lack of effect from cyclin A2 induction could be due to its degradation by APC/C. Thus, this degradation may reduce cyclin A to insufficient levels, preventing its impact on persister cell development. To further evaluate the impact of cyclin E and A overexpression on resistance development, we exposed palbociclib-resistant MCF-7 cells to the combination of palbociclib and INX-315. Both cyclin E1 and A2 overexpression significantly accelerated the development of resistance to the drug combination, with cyclin E1 having a more significant effect than cyclin A2 (Figure 7F). These results indicate that although CDK2 activity is targeted by CDK2i, the overexpression of cyclin E and A promotes resistance to the combination of CDK4/6i and CDK2i. Due to cyclin A degradation by APC/C in quiescent cells, cyclin E may play a more critical role in driving resistance to this drug combination.

## Discussion

The combination therapy of CDK4/6i and ET has revolutionized the treatment landscape for HR^+^/HER2^−^ breast cancer (48–55). Despite the initial efficacy of these treatments, the inevitable emergence of drug resistance remains a significant challenge, mainly as there is no established second-line therapy. Our findings demonstrate that sustained CDK4/6i treatment in drug-resistant cells results in ineffective Rb inactivation, significantly delaying E2F activation and G1-phase progression (Figure 7G). Concurrent ET further suppresses G1 progression by inhibiting c-Myc-mediated E2F amplification, intensifying the blockade of cell-cycle progression. Additionally, we observed that CDK2i is only effective at suppressing CDK2 activity and the growth of drug-resistant cells when combined with CDK4/6i. Notably, overexpression of cyclin E and A fosters resistance to this drug combination. These insights suggest the advantages of continuing CDK4/6i therapy beyond disease progression, both in vitro and in vivo. Moreover, it aligns with previous reports highlighting the importance of dual targeting CDK4/6 and CDK2 (26–30).

Our data indicate that maintaining both CDK4/6i and ET synergistically decelerates cell-cycle progression in drug-resistant cells by further delaying CDK2 activation kinetics and the G1/S transition without affecting the S and G2 phases. This dual effect stems from CDK4/6i causing suboptimal Rb inactivation while ET suppresses the global transcription amplifier c-Myc, collectively leading to diminished E2F transcriptional activity. As a result, this reduced E2F activity lowers the expression of critical cell-cycle genes, such as cyclin E and A, extending the time needed for CDK2 activation. Given that CDK2 plays an essential role in initiating and advancing DNA replication (56,57), its delayed activation significantly prolongs the G1/S transition. Moreover, CDK2 activation also contributes to Rb phosphorylation and inactivation. High CDK2 activity is required to phosphorylate Rb, and CDK2-mediated Rb phosphorylation is tightly coupled with DNA replication timing (7,40). Thus, upon Rb phosphorylation by CDK2 at the G1/S transition, drug-resistant cells may effectively proceed through the cell cycle even under continued CDK4/6i treatment.

Clinical trials evaluating the efficacy of sustained CDK4/6i therapy predominantly use PFS as the primary endpoint (18–22). However, our findings suggest that drug-resistant tumors continue to proliferate despite CDK4/6i maintenance. Consequently, maintaining CDK4/6i appears to slow tumor growth rather than completely arrest it. This underscores the need for clinical trials to consider overall survival and tumor progression rates as more appropriate endpoints for assessing the true benefits of sustained CDK4/6i therapy. Furthermore, the distinct polypharmacology profiles among CDK4/6i (58), with ribociclib being the most specific and abemaciclib the least, may explain the varying therapeutic outcomes observed among these inhibitors (20,59).

Maintaining CDK4/6i treatment post-disease progression may be particularly beneficial for approximately 70% of patients who do not acquire new genetic mutations (60). However, it is important to recognize that resistance to CDK4/6i often arises from mutations in genes associated with mitogenic or hormone-signaling pathways (60–64). These include mutations in *PIK3CA*, *ESR1*, *FGFR1*–*3*, and *HER2*, which have been linked to increased c-Myc expression (43,65,66). Additionally, recent studies have identified *FAT1* mutations as a driver of CDK4/6i resistance (35,67). These resistance mutations may reduce the efficacy of maintaining CDK4/6i and ET therapy. Moreover, approximately 4.7% of HR^+^/HER2^−^ breast cancer patients exhibit *Rb* mutations (60,62), making CDK4/6i treatment unlikely to be effective, thus making its continuation unadvisable in these cases.

In conclusion, our study offers significant mechanistic insights that support the continued use of CDK4/6i and ET in patients with HR^+^/HER2^−^ breast cancer after disease progression. Our findings also emphasize the necessity of combining CDK2i with CDK4/6i to enhance therapeutic efficacy, reinforcing conclusions from previous studies (26–29,44). Moreover, we underscore the critical role of cyclin E overexpression in driving resistance to the combination of CDK4/6i and CDK2i, which may inform future strategies to overcome therapeutic resistance in breast cancer.

## Materials and Methods

### Cell culture

MCF-7 (ATCC, HTB-22), CAMA1 (ATCC, HTB-21), and MDA-MB-231 (ATCC, CRM-HTB-26) cells were cultured in Dulbecco’s Modified Eagle Medium (DMEM; Genesee Scientific, 25–500) supplemented with 10% Fetal Bovine Serum (FBS; Gibco, A3160601). T47D (ATCC, HTB-133) cells were cultured in RPMI-1640 (Genesee Scientific, 25–206) with 10% FBS. All cell lines were incubated at 37 °C in a humidified atmosphere with 5% CO_2_. All cell lines were routinely tested and confirmed to be mycoplasma contamination-free.

### Drugs and Chemicals

Palbociclib (Selleck Chemicals, S1116), INX-315 (MedchemExpress, HY-162001), and fulvestrant (Selleck Chemicals, S1191) were dissolved in DMSO (Sigma-Aldrich, D2438) for in vitro experiments. For in vivo experiments, palbociclib (MedChemExpress, HY-50767), ribociclib (MedChemExpress, HY-15777), and abemaciclib (MedChemExpress, HY-16297A) were prepared in a solution of corn oil (Spectrum Chemical MFG, CO136) with 10% DMSO. Additional reagents, including 5-Ethynyl-2'-Deoxyuridine (EdU; Sigma-Aldrich, 900584) and AFDye-647 picolyl azide (Click Chemistry Tools, 1300) were used to measure cells undergoing DNA replication.

### Antibodies

The following antibodies were employed from Cell Signaling Technology: Rb (9309), Phosphorylated Rb (Ser807/811) (8516), and c-Myc (5605). Additional antibodies were obtained from Abcam for CDK6 (ab124821), Cyclin E (ab32103), and cyclin A (ab32386), and from BD Biosciences for Rb (554136). BD Biosciences Rb antibody was used to visualize Rb protein in cells expressing CDK4/6 sensor. Secondary antibodies from Thermo Scientific included Alexa Fluor 488 goat anti-mouse (A32723) and Alexa Fluor 568 goat anti-rabbit (A11036). For immunoblotting, we used secondary antibodies from LI-COR Biosciences: goat anti-mouse IR Dye 800CW (926–32210) and goat anti-rabbit IR Dye 680RD (926–68071).

### DNA constructs and cell line generation

All DNA constructs used in this study were generated as described previously (7,31,38,39,68). Briefly, using Gibson assembly, constructs including H2B-iRFP670-p2a-mCerulean-Cdt1 (a.a.1–100) (Addgene, 223965), H2B-iRFP670-p2a-mCerulean-Geminin (a.a.1–110) (Addgene, 223959), and DHB (a.a.995–1087)-mVenus-p2a-mCherry-Rb (a.a.886–928) (Addgene, 126679) were inserted into pLenti-IRES vectors encoded with puromycin, blasticidin, or neomycin selection markers. Doxycycline-inducible c-Myc, cyclin E1, and cyclin A2 were created by inserting PCR products into the lentiviral pCW57.1 vector (Addgene, 50661) following NheI and BamHI restriction digestion. Stable cell lines were established through lentiviral transduction. For virus production, lentiviral plasmids encoding the genes of interest were transfected into HEK-293T cells along with packaging plasmids pMDLg/pRRE (Addgene, 12251), pRSV-Rev (Addgene, 12253), and envelope plasmid pCMV-VSV-G (Addgene, 8454), using polyethyleneimine transfection reagent in Opti-MEM (Thermo Scientific, 31985070). Following 72 and 96 hr of incubation, the viral supernatant was collected, pooled, and centrifuged at 1,200 rpm for 5 minutes, followed by filtration through a 0.45 μm filter (Millipore, SLHA033SB). The filtered supernatant was then concentrated using an ultra-centrifugal filter (Millipore, UFC910024) at 4,000 rpm for 10 minutes. These concentrated viruses were stored at −80°C until further use. Cells were infected with virus in the presence of 5 μM polybrene for two days and subsequently selected either through antibiotic selection with 1 μg/ml puromycin (InvivoGen, ant-pr), 10 μg/ml blasticidin (InvivoGen, ant-bl), or 800 μg/ml neomycin (Thermo Scientific, BP673–5), or alternatively by sorting cells based on their introduced fluorescent protein using an Influx cell sorter.

### CDK6 and Rb knockout cell lines

Rb knockout in MCF-7 cells was previously described and validated (38). To establish CDK6 knockout, MCF-7 cells were transfected with separate crRNA:tracrRNA-ATTO550 plus CRISPR-Cas9 ribonucleoprotein complexes. Successfully transfected cells, identified by ATTO550 fluorescence, were isolated into single cells by fluorescence-activated cell sorting and then grown into colonies. Knockouts were confirmed by immunoblot. Two pooled CRISPR-Cas9 guides were used to knockout CDK6 gene:

- CDK6 gRNA 1: 5’-GACCACGUUGGGGUGCUCGAGUUUUAGAGCUAUGCU-3'
- CDK6 gRNA 2: 5’-CUGGACUGGAGCAAGACUUCGUUUUAGAGCUAUGCU-3'

### Drug-resistant cell lines

To induce resistance to CDK4/6i, breast cancer cells were subjected to chronic exposure to palbociclib (1 μM) or a combination of palbociclib (1 μM) and fulvestrant (500 nM) over 1–2 months. The cell culture media containing the drugs was refreshed every 2–3 days throughout the experimental period. Resistance development was confirmed by assessing drug sensitivity through titration of various drug concentrations and calculating the half-maximal inhibitory concentration (IC50) based on the proportion of S-phase cells compared to drug-naïve controls.

### Live-cell reporters

Histone 2B (H2B) tagged with iRFP670 was used to define nuclear regions and track individual cells. Live-cell reporters were employed to monitor CDK activity and cell-cycle transitions. Fluorescently tagged Cdt1 degron (a.a.1–100) (33) was used to detect cell-cycle phase transitions. Fluorescent geminin protein was used to track S-phase entry. CDK4/6 and CDK2 kinase translocation reporters (KTRs) (31,32) were used to monitor the phosphorylation state of specific substrates. The CDK4/6 substrate was the C-terminal domain of Rb (a.a.886–928), and the CDK2 substrate was DNA helicase B (a.a.994–1087). Fluorescence translocation between the nucleus and cytoplasm provided a quantitative readout of CDK activity. The cytoplasmic/nuclear fluorescence intensity ratio was used to represent kinase activity. Since the CDK4/6 KTR contains a degenerate CDK2 substrate motif from the CDK2 KTR and thus detects activities measured by the CDK2 sensor during the S and G2 phases, we applied a correction factor derived from a linear regression based on the CDK2 sensor, as described previously (31,38,68). CDK4/6 activity was adjusted by subtracting a calculated fraction of the CDK2 KTR signal, as follows:

MCF-7: CDK4/6 activity = CDK4/6 reporter activity – (0.41 × CDK2 reporter activity)

MDA-MB-231: CDK4/6 activity = CDK4/6 reporter activity – (0.35 × CDK2 reporter activity)

### Immunofluorescence and mRNA fluorescence in situ hybridization (FISH)

Cells were seeded in glass-bottom 96 well plates (Cellvis, P96–1.5H-N) at least one night prior to each experiment. The percentage of S-phase cells was determined using EdU incorporation. Cells were incubated with EdU (10 μM) for 15 minutes at 37°C, followed by fixation with 4% formaldehyde (Thermo Scientific, 28906) containing 10 mM HEPES (Sigma-Aldrich, H3537) in PBS for 15 minutes at room temperature. Permeabilization was then performed using 0.2% Triton X-100 (Sigma-Aldrich, T8787) in PBS (PBS-T) for 15 minutes. Visualization of EdU incorporation was achieved using a click reaction in 2 mM CuSO_4_ (Sigma-Aldrich, C1297), 20 mg/ml sodium ascorbate (Sigma-Aldrich, A4034), and 3 μM Alexa Fluor Dye 647 picolyl azide in Tris-Buffered Saline (TBS) (Sigma-Aldrich, T6066) (pH 8.3) for 15 minutes. For immunofluorescence staining, cells were incubated in blocking buffer (10% FBS, 1% BSA, 0.1% Triton X-100, and 0.01% NaN_3_ in PBS) for 1 hour. Cells were then incubated with primary antibodies for Phospho-Rb (Cell Signaling Technology, 8516), Rb (BD Biosciences, 554136), and c-Myc (Cell Signaling Technology, 5605), diluted 1:2000 in blocking buffer, overnight at 4°C. The following day, Alexa Fluor 488- or 568-conjugated secondary antibodies were applied at 1:2000 dilution in blocking buffer for 1 hour at room temperature. DNA was stained with Hoechst 33342 dye (Thermo Scientific, 62249) (10 mg/ml), diluted in PBS (1:10000) for 15 minutes at room temperature prior to imaging. Cells were washed with PBS between each step and stored in PBS prior to imaging. For mRNA FISH, the ViewRNA ISH Cell Assay Kit (Thermo Scientific, QVC0001) was used along with a probe targeting E2F1 (Thermo Scientific, VA1–12108-VC). Following live-cell imaging, cell fixation and permeabilization were conducted as described in the immunofluorescence section. The fluorescent protein signal was then photobleached using 3 % H_2_O_2_ (Sigma-Aldrich, H1009) with 20 mM HCl (Sigma-Aldrich, 320331) in PBS for 2 hours at room temperature. Probe hybridization, amplification, and Alexa Fluor 555 labeling were carried out following the manufacturer’s protocols.

### Growth curve measurement

To assess overall cell proliferation, 5,000 cells were seeded into each well of a 24-well plate (Fisher Scientific, FB012929). Cells were harvested at the indicated time points by trypsinization, resuspended in 1 ml growth media, and manually counted using a hemocytometer (Hausser Scientific, HS-3510). Cells were then replated as needed for subsequent time points.

### Live and Fixed cell and Tumor tissue image acquisition

Cells were seeded and maintained at 30–80% confluency during experiments in 96-well glass-like polymer bottom plates (Cellvis, P96–1.5P). Phenol red-free media was used to minimize background fluorescence. Cell imaging was conducted using a microscope mounted onto an inverted Eclipse Ti-2 body (Nikon), and multichannel fluorescent images were captured with a Hamamatsu ORCA-Fusion camera. Acquisition used either a 10× (Nikon CFI Plan Apo Lambda, 0.45 NA, no binning) or a 20× (Nikon CFI Plan Apo Lambda, 0.75 NA, 2×2 binning) objective. Live-cell imaging was performed in a 37 °C humidified chamber with 5% CO_2_, capturing 3 sites per well at 12-min intervals. 9 (10× objective) or 32 (20× objective) sites per well were collected for fixed-cell imaging. Total light exposure time was kept under 500 ms for each time point. mRNA FISH images were acquired with 1.5 μm z-stack intervals. Whole tumor cross-sections were imaged using an Axio Observer 7 microscope with Apotome 2 (Zeiss) and a Hamamatsu ORCA-Flash 4 camera, using a 10× (Zeiss Plan-Apochromat, 0.45 NA, no binning) objective at a 1.5 μm z-stack interval.

### Image processing and analysis

Live- and fixed-cell image processing and analysis were conducted using custom MATLAB scripts (MathWorks, R2021a). To reduce biased light illumination, flat-field correction was applied. In fixed-cell analysis, nuclei were segmented based on the Hoechst signal according to a threshold of histogram curvature. For live-cell analysis, cell segmentation was performed using the H2B-iRFP670 signal and a Laplacian of Gaussian blob detector. A marker-based watershed algorithm was then employed to separate neighboring nuclei. To correct for background signal, the 50^th^ percentile of pixels in the remaining background was subtracted after masking nuclei. In the analysis transitioning from live to fixed cells, cells were initially segmented and then aligned between the fixed cell data and the last frame of the live-cell experiment. A deflection-bridging algorithm was utilized to track cell movement over time during live-cell acquisition. Mitotic events were identified by isolating two daughter cell nuclei adjacent to a prior nucleus, with H2B signals approximately 45–55% of the mother cell’s nuclear signal. In live-cell analysis, cytoplasmic signal determination involved measuring the median pixel intensity within a ring positioned between 0.65–3.25 μm outside the nuclear perimeter. Cells with overlapping cytoplasmic rings were excluded from the final analysis. For RNA FISH, the whole cell area was estimated by creating a mask including and extending 50 μm from the nucleus. Overlapping cells with adjacent cell nuclei were excluded from the analysis. Raw images underwent a top hat filter with a 4 μm radius circular kernel to create the FISH puncta mask. Tissue image analysis was performed using ImageJ software.

### Immunoblot

Cells were rinsed with ice cold PBS before incubation in 300 μl CHAPS lysis buffer (50 mM Tris-HCl, 150 mM NaCl, 1 mM EDTA, 10 mM NEM, 0.3% CHAPS, 1 mM PMSF) supplemented with 1× Halt Protease inhibitor (Thermo Scientific, 1861279) and 1× Pierce phosphatase inhibitor (Roche, 4906845001) for 5 minutes on ice. Lysates were collected and then spun at 4°C for 10 minutes at 14,000 RCF, and supernatants were used for subsequent experiments. Protein concentration was determined by spectrophotometric interpolation after standard curve construction using the Pierce 660 nm protein assay reagent (Thermo Scientific, 22660) according to the manufacturer’s instructions. 12 μg of protein were incubated in LDS sample buffer (Thermo Scientific, NP0007) at 70°C for 10 minutes before separation on NuPAGE 4–12% Bis-Tris gel (Thermo Scientific, NP0322BOX). Protein was then transferred to a 0.45 μm mini PVDF transfer membrane (Bio-Rad, 1620174) using a Bio-Rad Trans-Blot Turbo system (Bio-Rad, 1704150) and 1× Trans-Blot Transfer Buffer (Bio-Rad, 10026938). Membranes were then blocked with Intercept Blocking Buffer (LI-COR, 927–60001) at room temperature for 1 hour. Immunoblots were subsequently incubated with either Rb (Cell Signaling Technology, 9309), Phospho-Rb (Cell Signaling Technology, 8516), CDK6 (Abcam, ab124821) or Beta-actin (Cell Signaling Technology, 3700S) primary antibody diluted 1:1000 in Intercept Blocking Buffer overnight at 4°C. After primary incubation, membranes were washed with TBS-T (20 mM Tris, 150 mM NaCl, 0.1% Tween 20, pH 7.5) and incubated in either goat-anti mouse IR Dye 800CW (LI-COR, 926–32210) or goat-anti rabbit IR Dye 680RD (LI-COR, 926–68071) at 1:2000 in Intercept Blocking Buffer for 2 hours at room temperature. After rinsing with TBS-T, membranes were visualized using the LI-COR Odyssey Infrared Imaging System. Immunoblot images were processed with ImageStudio Lite Ver5.2.

### RNAseq

RNA isolation was performed using the GeneJET RNA Purification Kit (Thermo Scientific, K0732) according to manufacturer’s instruction. After trypsinization, cells were rinsed with PBS then lysed in suspension using 600 μL of the supplied lysis buffer supplemented with 14.3M β-mercaptoethanol (Bio-Rad, 1610710). After homogenization and addition of 360 μL 100% ethanol (Fisher Scientific, BP28184), RNA was isolated and collected by centrifugation before final elution with DNase/RNase-free distilled water (Thermo Scientific, 10977–015). RNA sequencing was then performed by Azenta Life Sciences. Statistical analysis of count matrix was performed using DESeq2 R package (v1.44.0) (69). Log2 fold-change shrinkage was applied to improve effect size estimates using the 'apeglm' method (70). Gene sets were obtained from the Molecular Signatures Database (Hallmark, C2, C5) utilizing the msigdbr package (version 7.5.1). Heatmaps were created with the ComplexHeatmap package (71). Gene set enrichment analysis was conducted with hallmark annotations from MSigDB using the R package clusterProfiler (v4.12.6). Ranked gene lists were input with a maximum gene set size of 500, and significance was evaluated through 10,000 permutations. Terms were considered significantly enriched if their *P* value was below 0.05 and their False Discovery Rate (FDR) was under 0.25.

### Animal

All experiments were conducted in accordance with NIH guidelines and protocols approved by the Institutional Animal Care and Use Committee (IACUC) at Columbia University Irving Medical Center. Six-week-old female Foxn1−/−J:NU mice (The Jackson Laboratory, 007850) were housed in a barrier facility with ad libitum access to food and water on a 12-hour light-dark cycle. Mice were regularly monitored in compliance with institutional guidelines for ethical endpoints.

### Xenograft experiments

Female J:NU mice were anesthetized with ketamine (45 mg/kg) plus xylazine (5 mg/kg) by intraperitoneal injection. Mice were then orthotopically injected with drug-naïve MCF-7 cells (8×10^6^ cells per mouse) suspended in 1:1 ratio of PBS and Geltrex LDEV-Free Reduced Growth Factor Basement Membrane Matrix (Thermo Scientific, A1413201) directly into the abdominal mammary fat pad. Tumors were measured biweekly using a digital caliper (volume = [width^2^ × length] × 1/2). Once tumor volumes reached ~100 mm^3^, mice began daily treatment with palbociclib (50 mg/kg) by oral gavage. When tumors reached between 145–155 mm^3^, mice were randomly assigned to 1) discontinuation of treatment, 2) daily palbociclib (50 mg/kg) treatment, 3) daily abemaciclib (50 mg/kg) treatment, or 4) daily ribociclib (200 mg/kg) treatment for 32 days. Upon treatment completion, mice were anesthetized by ketamine/xylazine, then perfused with 1% formaldehyde before tumor extraction.

### Immunohistochemistry

After vascular perfusion with 1% formaldehyde in PBS, tumors were fixed in 1% formaldehyde at 4°C for 1 hour. Following fixation, tissues were washed with PBS-T and incubated in 30% sucrose at 4°C overnight. Samples were embedded in optimal cutting temperature compound (Fisher Scientific, 23–730-571), frozen at -80°C, and sectioned into 40 μm sections onto Superfrost Plus microscope slides (Fisher Scientific, 12–550-15). Tumor sections were rehydrated in PBS-T and subsequently blocked with 5% normal goat serum (The Jackson Laboratories, 005–000-121) in PBS-T for 1 hour at room temperature. The sections were then incubated with recombinant anti-Rb monoclonal antibody (Abcam, ab181616) in 5% normal goat serum in PBS-T at 1:1000 overnight at room temperature. Following washing with PBS-T, the sections were incubated with Alexa Fluor 488 goat anti-rabbit (Thermo Scientific, A32731) at 1:500 in PBS-T at room temperature for 4 hours. DNA was counterstained with DAPI (Sigma-Aldrich, D9542) 1:500 in PBS-T for 10 minutes, then mounted with Fluoromount-G (Invitrogen, 00–4958-02) for image acquisition.

### Statistics and Reproducibility

Statistical analyses were performed using GraphPad Prism 10.2.0. For parametric data, we utilized unpaired two-tailed Student’s *t*-tests or one-way ANOVA with Tukey’s post hoc tests, as appropriate, to assess pairwise differences. When comparing two-paired groups, paired two-tailed Student’s *t*-tests were employed. Statistical test results are reported in the figure legends and detailed in Supplementary Table 1. All experiments were conducted in at least two independent experiments.

## Supplementary Material

Supplement 1
