# Supplementary material for: Therapeutic benefits of maintaining CDK4/6 inhibitors and incorporating CDK2 inhibitors beyond progression in breast cancer
Source: bioRxiv. 2024 Nov 15:2024.11.11.623139. Preprint. [Version 1] doi: 10.1101/2024.11.11.623139 (PMC11601343; doi:10.1101/2024.11.11.623139)
Supplement: Supplementary file 1 [file NIHPP2024.11.11.623139v1-supplement-1.pdf]

bioRxiv preprint doi: https://doi.org/10.1101/2024.11.11.623139; this version posted November 15, 2024. The copyright holder for this preprint (which was not certified by peer review) is the author/funder, who has granted bioRxiv a license to display the preprint in perpetuity. It is made available under aCC-BY 4.0 International license.

Supplementary Figure 1.

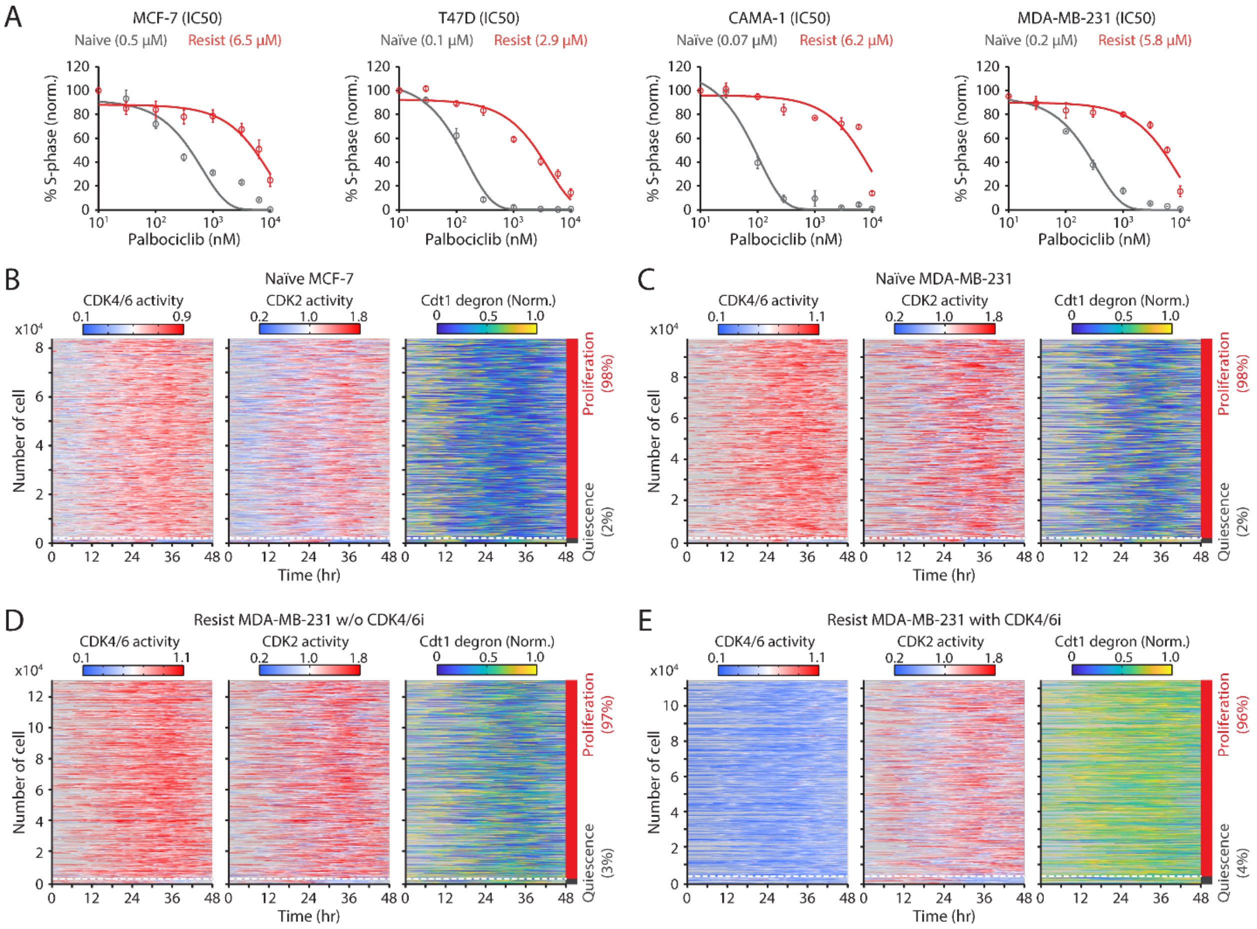

Validation of drug resistance and visualization of cell-cycle progression.

(A) Dose-response curves of breast cancer cells treated with palbociclib (10 nM - 10 μM) for 48 hr. Data are shown as mean ± SEM (*n* = 3 biological replicates). Solid lines represent sigmoidal best-fit curves. (B-E) Heatmaps of single-cell traces for CDK4/6 (left) and CDK2 (middle) activities, and Cdt1-degron intensity (right) in various conditions: drug-naïve MCF-7 (B) and MDA-MB-231 (C) cells, and drug-resistant MDA-MB-231 cells without (D) or with (E) continuous palbociclib (1μM) treatment. Proliferating cells were identified based on CDK2 activity (>1 for more than 2 hr between 30 and 48 hr).

bioRxiv preprint doi: https://doi.org/10.1101/2024.11.11.623139; this version posted November 15, 2024. The copyright holder for this preprint (which was not certified by peer review) is the author/funder, who has granted bioRxiv a license to display the preprint in perpetuity. It is made available under aCC-BY 4.0 International license.

Supplementary Figure 2.

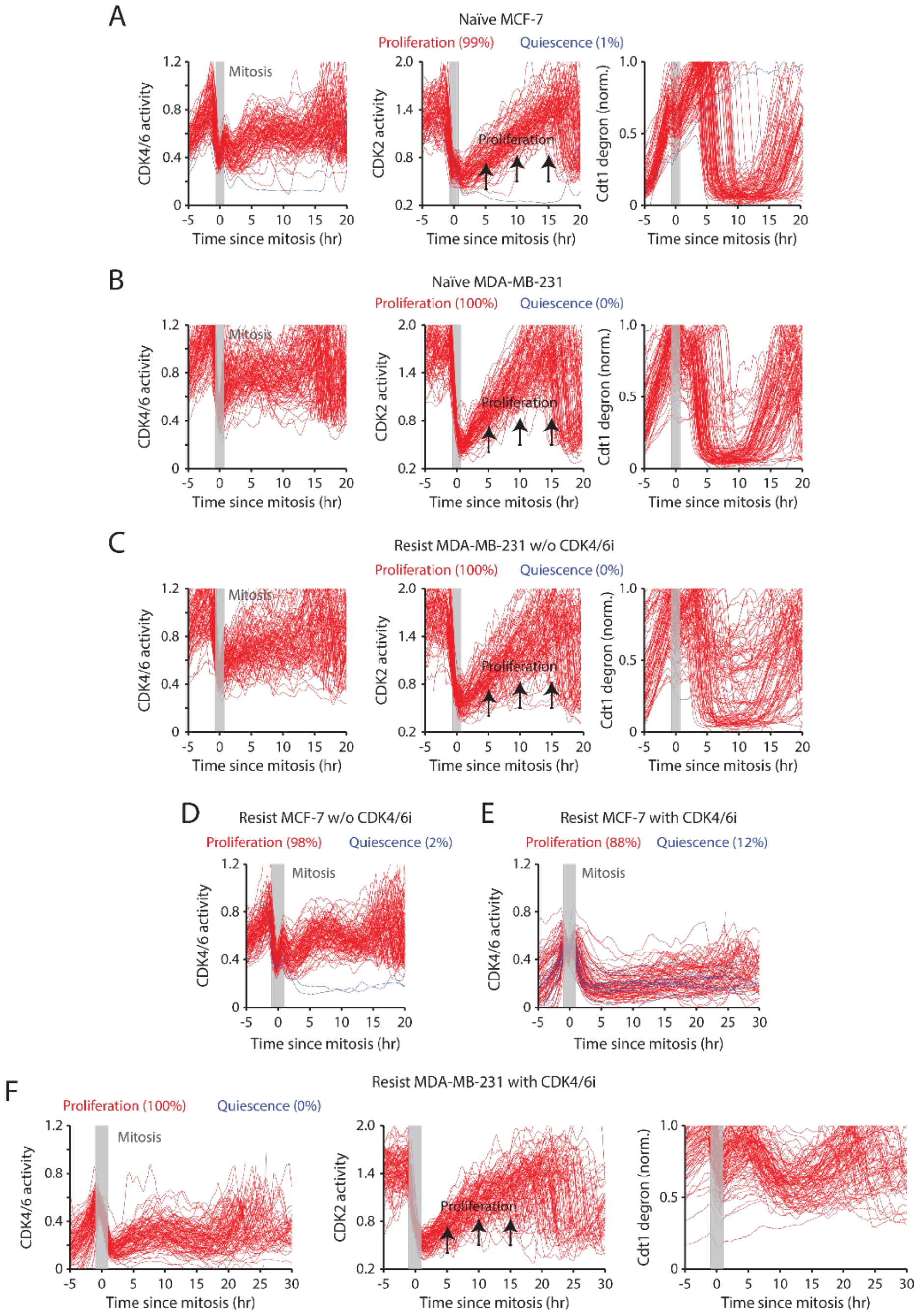

Slow cell-cycle progression in drug-resistant cells continuously treated with CDK4/6i. (A-C) Single-cell traces of CDK4/6 (left) and CDK2 (middle) activities and Cdt1-degron intensity (right) aligned by mitosis in drug-naïve MCF-7 (A) and MDA-MB-231 (B) cells and drug-resistant MDA-MB-231 cells without CDK4/6i treatment (C). The time of mitosis is marked in gray. **(**D-E) Single-cell trace of CDK4/6 activity corresponding to Figure 1G (D) and 1H (E). (F) Single-cell

bioRxiv preprint doi: https://doi.org/10.1101/2024.11.11.623139; this version posted November 15, 2024. The copyright holder for this preprint (which was not certified by peer review) is the author/funder, who has granted bioRxiv a license to display the preprint in perpetuity. It is made available under aCC-BY 4.0 International license.

traces of CDK4/6 (left) and CDK2 (middle) activities and Cdt1-degron intensity (right) aligned by mitosis in drug-resistant MDA-MB-231 cells with continuous palbociclib (1 μM) treatment.

bioRxiv preprint doi: https://doi.org/10.1101/2024.11.11.623139; this version posted November 15, 2024. The copyright holder for this preprint (which was not certified by peer review) is the author/funder, who has granted bioRxiv a license to display the preprint in perpetuity. It is made available under aCC-BY 4.0 International license.

Supplementary Figure 3.

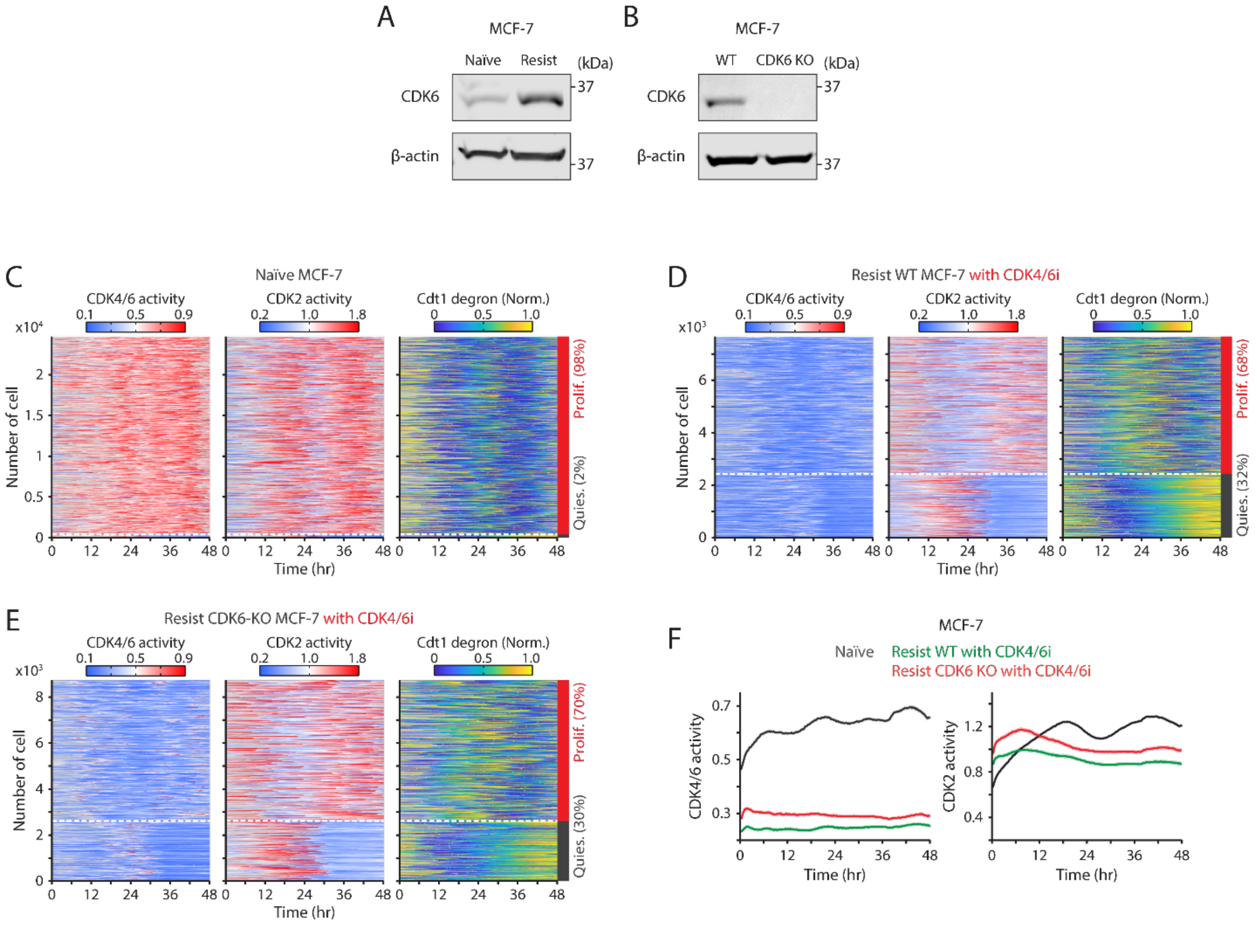

Potential non-canonical role of CDK6 in promoting CDK4/6i resistance.

(A-B) Immunoblot showing CDK6 and β-actin expression in drug-naïve and palbociclib-resistant cells (A) and WT and CDK6-KO cells (B). (C-E) Heatmaps of single-cell traces for CDK4/6 (left) and CDK2 (middle) activities, and Cdt1-degron intensity (right) in drug-naïve (C) and drug-resistant WT (D) and CDK6-KO (E) cells with continued palbociclib (1 μM) treatment. Proliferating cells were identified based on CDK2 activity (>1 for more than 2 hr between 30 and 48 hr). (F) Averaged traces of CDK4/6 (left) and CDK2 (right) activities. Data are shown as mean ± 95% confidence intervals (n > 7,000 cells/condition).

bioRxiv preprint doi: https://doi.org/10.1101/2024.11.11.623139; this version posted November 15, 2024. The copyright holder for this preprint (which was not certified by peer review) is the author/funder, who has granted bioRxiv a license to display the preprint in perpetuity. It is made available under aCC-BY 4.0 International license.

Supplementary Figure 4

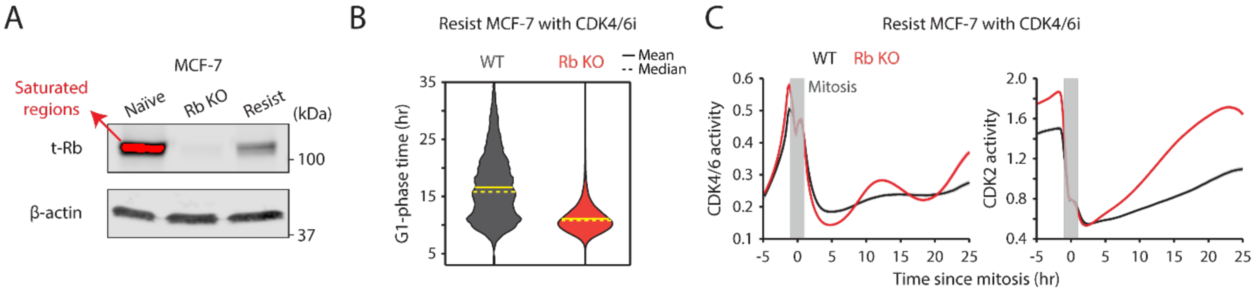

Incomplete Rb loss mediates the extended G1-phase progression.

(A) Immunoblot showing total Rb and β-actin in WT, Rb-KO, and palbociclib-resistant cells. (B) G1-phase duration in drug-resistant WT and Rb-KO cells with continued palbociclib (1 μM) treatment. Solid and dashed yellow lines represent mean and median, respectively (*n* > 4,500 cells). (C) Averaged CDK4/6 (left) and CDK2 (right) activities aligned by mitosis in drug-resistant WT and Rb-KO cells with continued palbociclib (1 μM) treatment (*n* > 7,500 cells).

bioRxiv preprint doi: https://doi.org/10.1101/2024.11.11.623139; this version posted November 15, 2024. The copyright holder for this preprint (which was not certified by peer review) is the author/funder, who has granted bioRxiv a license to display the preprint in perpetuity. It is made available under aCC-BY 4.0 International license.

Supplementary Figure 5.

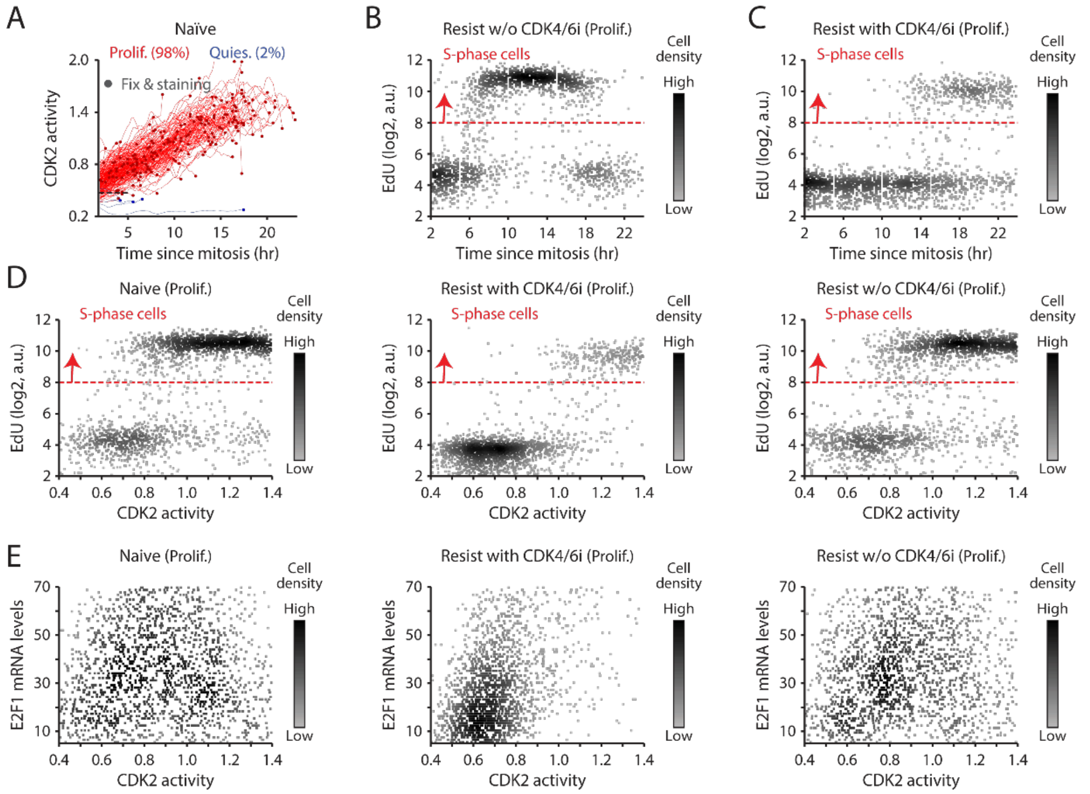

Slow and heterogeneous G1/S transition in drug-resistant cells maintained with CDK4/6i treatment.

(A) Single-cell traces showing CDK2 activity aligned by mitosis in proliferating (red) and quiescent (blue) drug-naïve MCF-7 cells. Circles indicate the time of fixation and staining (n = 200 cells). (B-C) Scatterplot of EdU intensity against time since mitosis in drug-resistant cells without (B) and with (C) continuous palbociclib (1 μM) treatment. Red dotted line indicates the S-phase threshold (n = 2,000 cells/condition). (D-E) Scatterplot of EdU intensity (D) and E2F1 mRNA levels (E) against CDK2 activity (n = 2,000 cells/condition).

bioRxiv preprint doi: https://doi.org/10.1101/2024.11.11.623139; this version posted November 15, 2024. The copyright holder for this preprint (which was not certified by peer review) is the author/funder, who has granted bioRxiv a license to display the preprint in perpetuity. It is made available under aCC-BY 4.0 International license.

Supplementary Figure 6.

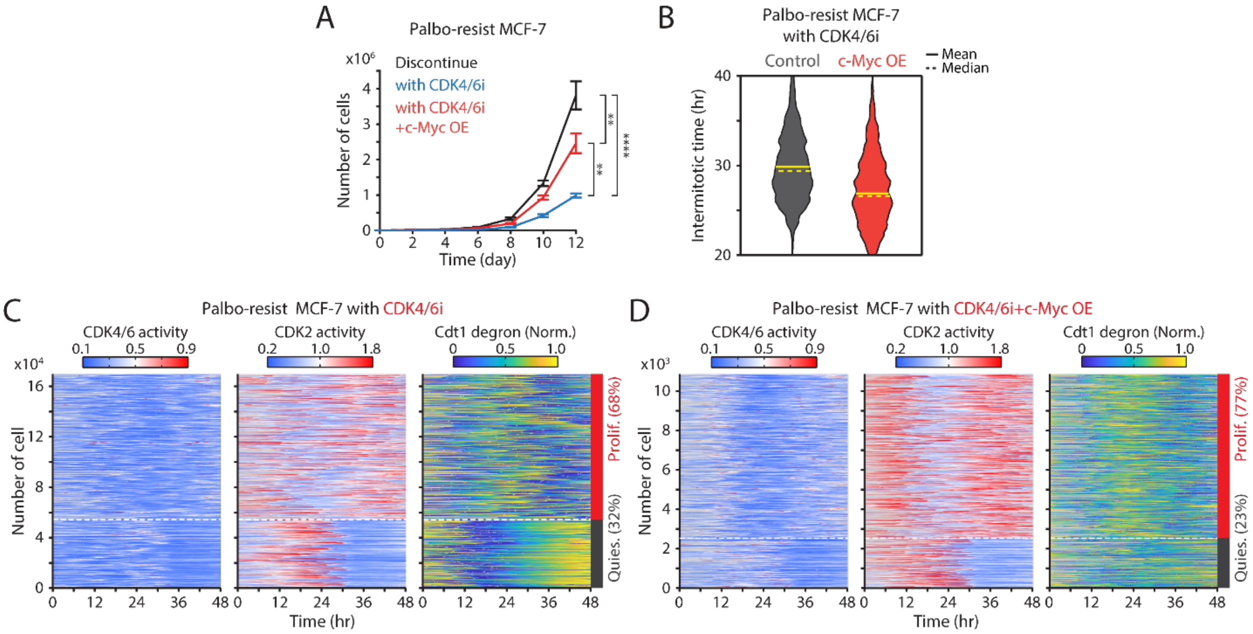

c-Myc overexpression facilitates CDK4/6i resistance by accelerating cell-cycle progression. (A) Growth curves of drug-resistant cells with treatment discontinuation or continuous palbociclib (1 µM) treatment, without or with c-Myc overexpression. Data are shown as mean ± SD (n = 3 biological replicates). Statistical significance was determined using one-way ANOVA with Tukey's post-hoc analysis (** $P < 0.01$, **** $P < 0.0001$). (B) Intermitotic time of drug-resistant cells without and with c-Myc overexpression. Solid and dashed yellow lines represent mean and median, respectively. (C-D) Heatmaps of single-cell traces for CDK4/6 (left) and CDK2 (middle) activities, and Cdt1-degron intensity (right) in drug-resistant cells undergoing continuous palbociclib (1 µM) treatment without (C) and with (D) c-Myc overexpression. Proliferating cells were identified based on CDK2 activity (>1 for more than 2 hr between 30 and 48 hr).

bioRxiv preprint doi: https://doi.org/10.1101/2024.11.11.623139; this version posted November 15, 2024. The copyright holder for this preprint (which was not certified by peer review) is the author/funder, who has granted bioRxiv a license to display the preprint in perpetuity. It is made available under aCC-BY 4.0 International license.

Supplementary Figure 7.

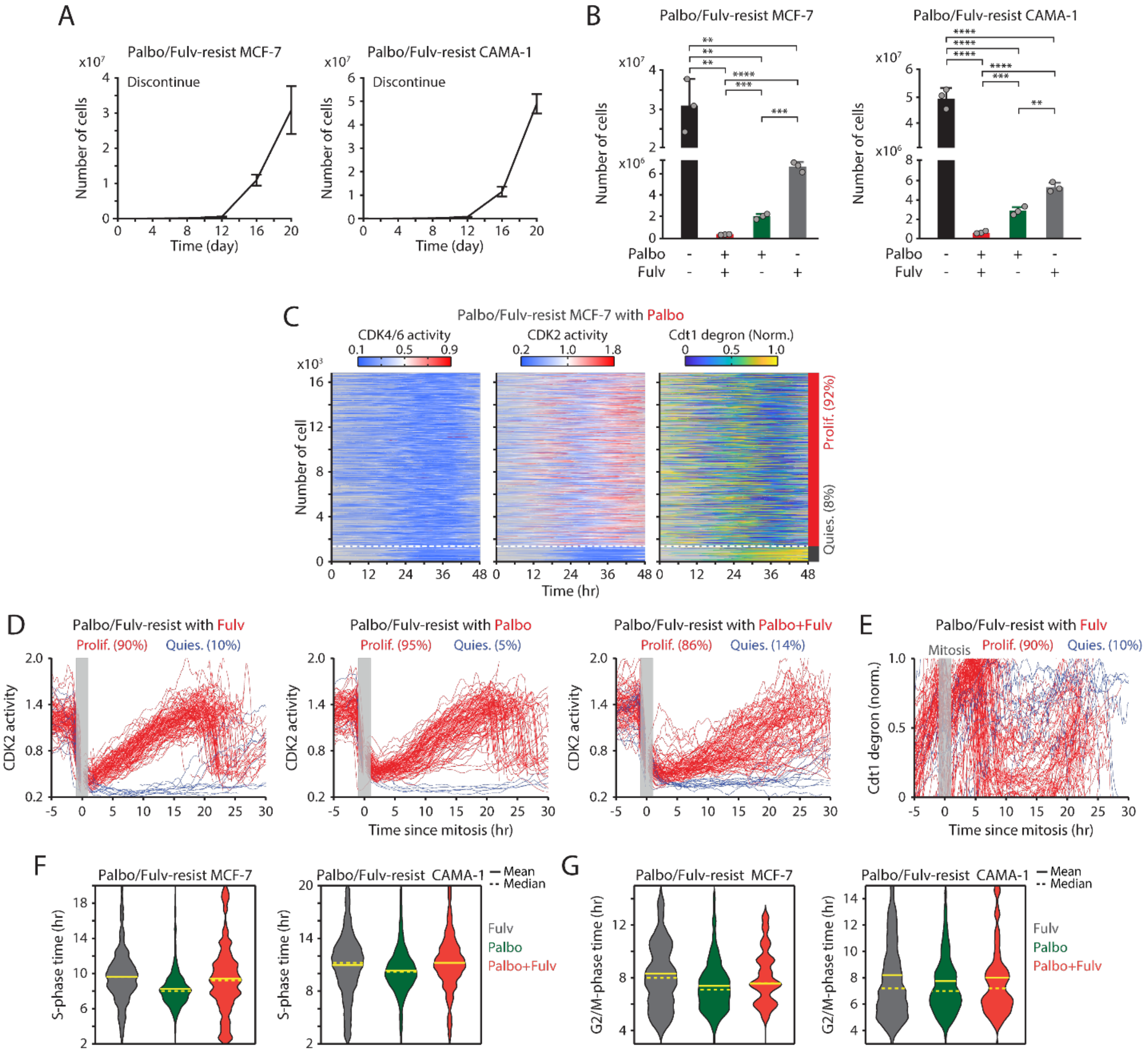

Maintaining CDK4/6i and ET synergistically suppresses the growth of drug-resistant cells.

(A) Growth curves of MCF-7 (left) and CAMA-1 (right) cells resistant to palbociclib and fulvestrant withdrawn from drug treatment. Data are shown as the mean ± SD (*n* = 3 biological replicates). (B) Cell numbers 20 days after treatment with DMSO, palbociclib (1 μM), fulvestrant (500 nM), or their combination. Data are shown as mean ± SD (*n* = 3 biological replicates). Statistical significance was determined with an unpaired *t*-test (** $P < 0.01$, *** $P < 0.001$, **** $P < 0.0001$). (C) Heatmaps of single-cell traces for CDK4/6 (left) and CDK2 (middle) activities, and Cdt1-degron intensity (right) in combination drug-resistant cells maintained with palbociclib (1 μM). Proliferating cells were identified based on CDK2 activity (>1 for more than 2 hr between

bioRxiv preprint doi: https://doi.org/10.1101/2024.11.11.623139; this version posted November 15, 2024. The copyright holder for this preprint (which was not certified by peer review) is the author/funder, who has granted bioRxiv a license to display the preprint in perpetuity. It is made available under aCC-BY 4.0 International license.

30 and 48 hr). (D) Single-cell traces of CDK2 activity aligned by mitosis in combination drug-resistant cells treated with continuous fulvestrant (500 nM) (left), palbociclib (1 μM) (middle), or their combination (right). Based on CDK2 activity, cells were classified into proliferation (red) or quiescence (blue). The time of mitosis is marked in gray. (E) Single-cell traces of Cdt1-degron intensity aligned by mitosis in combination drug-resistant cells treated with continuous fulvestrant (500 nM) alone. (F-G) Violin plots showing S-phase duration ($n > 220$ cells/condition) (F) and G2/M-phase duration ($n > 23$ cells/condition) (G) in MCF-7 (left) and CAMA-1 (right) cells. Solid and dashed yellow lines indicate mean and median, respectively.

bioRxiv preprint doi: https://doi.org/10.1101/2024.11.11.623139; this version posted November 15, 2024. The copyright holder for this preprint (which was not certified by peer review) is the author/funder, who has granted bioRxiv a license to display the preprint in perpetuity. It is made available under aCC-BY 4.0 International license.

Supplementary Figure 8.

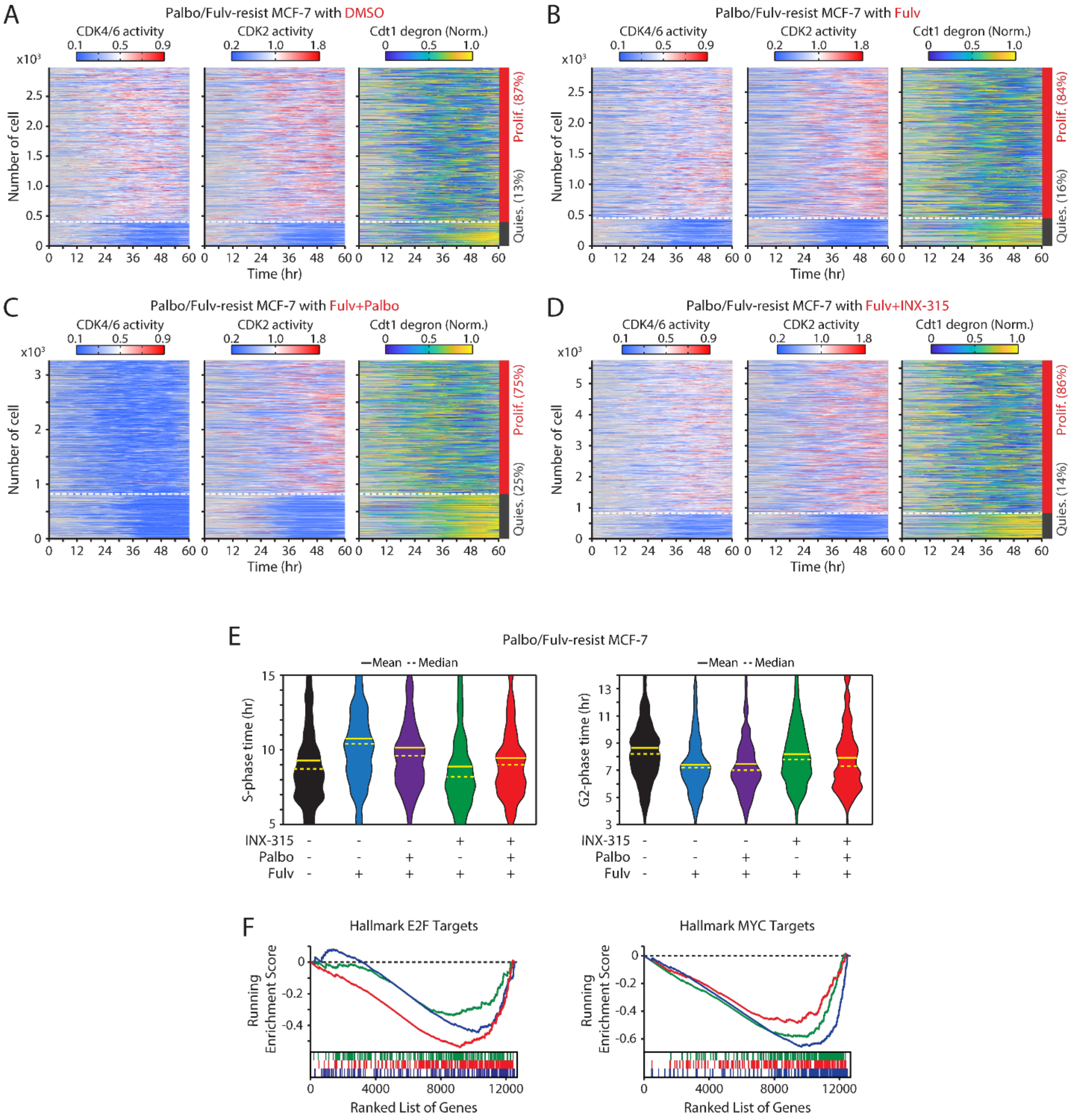

		E2F Targets		MYC Targets	
		NES	q-value	NES	q-value
Palbo + INX-315 + Fulv compared to	Discontinue	-1.375	0.0284	-2.381	0.0007
	INX-315 + Fulv	-2.451	0.0010	-2.200	0.0010
	Palbo + Fulv	-1.604	0.0025	-2.372	0.0009

Combining CDK2i with CDK4/6i and ET effectively suppresses the growth of drug-resistant cells.

(A-D) Heatmaps of single-cell traces for CDK4/6 (left) and CDK2 (middle) activities, and Cdt1-degron intensity (right) in drug-resistant cells under various conditions: treatment discontinuation

bioRxiv preprint doi: https://doi.org/10.1101/2024.11.11.623139; this version posted November 15, 2024. The copyright holder for this preprint (which was not certified by peer review) is the author/funder, who has granted bioRxiv a license to display the preprint in perpetuity. It is made available under aCC-BY 4.0 International license.

(A), fulvestrant (500 nM) alone (B), fulvestrant + palbociclib (1 μM) (C), or fulvestrant + INX-315 (100 nM) (D) over one week prior to imaging. Proliferating cells were identified based on CDK2 activity (>1 for more than 2 hr between 30 and 48 hr). (E) Violin plots showing S-phase duration (n > 400 cells/condition) (left) and G2/M-phase duration (n > 150 cells/condition) (right) in drug-resistant MCF-7 cells treated with the indicated drug. Solid and dashed yellow lines indicate mean and median, respectively. (F) GSEA plots for hallmark E2F (left) and MYC (right) target genes in palbociclib/fulvestrant-resistant MCF-7 cells treated with the triple combination, compared to other drug conditions.

bioRxiv preprint doi: https://doi.org/10.1101/2024.11.11.623139; this version posted November 15, 2024. The copyright holder for this preprint (which was not certified by peer review) is the author/funder, who has granted bioRxiv a license to display the preprint in perpetuity. It is made available under aCC-BY 4.0 International license.

575 **Supplementary Figure 9.**

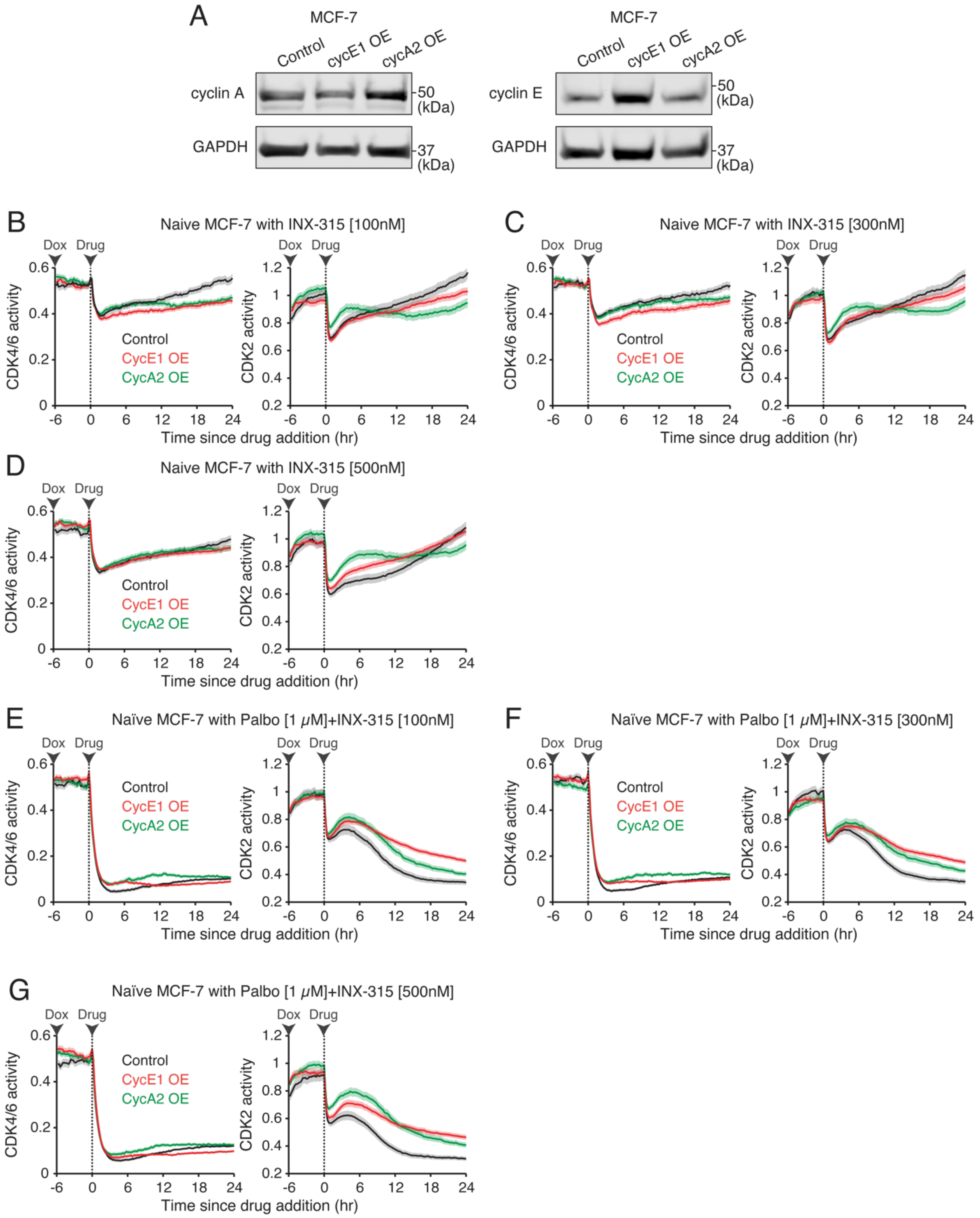

bioRxiv preprint doi: https://doi.org/10.1101/2024.11.11.623139; this version posted November 15, 2024. The copyright holder for this preprint (which was not certified by peer review) is the author/funder, who has granted bioRxiv a license to display the preprint in perpetuity. It is made available under aCC-BY 4.0 International license.

Cyclin E or A overexpression attenuates full CDK2 inhibition by CDK4/6i and CDK2i combination.

(A) Immunoblot showing the expression of GAPDH with cyclin A (left) or cyclin E (right) in MCF-7 cells with or without cyclin E1 or A2 overexpression. Cells were treated with doxycycline (500 nM) for 24 hr. (B-G) Averaged traces of CDK4/6 (left) and CDK2 (right) activities in MCF-7 cells with or without cyclin E1 and A2 overexpression. Cells were treated with indicated doxycycline (500 nM) 6 hr before the addition of indicated drugs. Data are shown as mean ± 95% confidence intervals (n > 850 cells/condition).

bioRxiv preprint doi: https://doi.org/10.1101/2024.11.11.623139; this version posted November 15, 2024. The copyright holder for this preprint (which was not certified by peer review) is the author/funder, who has granted bioRxiv a license to display the preprint in perpetuity. It is made available under aCC-BY 4.0 International license.

Supplementary Figure 10.

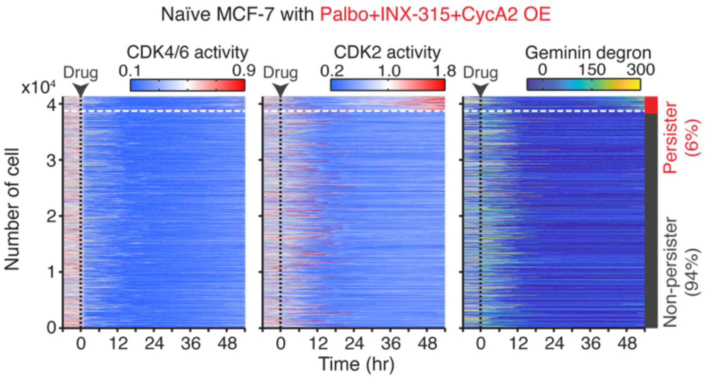

Cyclin E overexpression facilitates adaptation to CDK4/6i and CDK2i combination.

Heatmaps of single-cell traces for CDK4/6 (left) and CDK2 (middle) activities, and Geminin-
590 degron intensity (right) in drug-naïve MCF-7 cells overexpressing cyclin A and treated with palbociclib (1 μM) and INX-315 (100 nM). Proliferating cells were identified based on CDK2 activity (>1 for more than 2 hr between 30 and 48 hr).
